# Metabolic activation of pyruvate cycle by protocatechualdehyde triggers oxidative killing of ampicillin-resistant *Escherichia coli*

**DOI:** 10.1128/spectrum.02879-25

**Published:** 2026-03-18

**Authors:** Liting Cheng, Yue Liu, Yifei Ge, Shuhan Yang, Tao Yuan, Sufang Kuang

**Affiliations:** 1 Jiangxi Province Key Laboratory of Natural and Biomimetic Drugs Research, College of Pharmacy, Jiangxi Normal University 12642, Nanchang, Jiangxi, China; 2 College of Life Sciences, Jiangxi Normal University 12642, Nanchang, China; University of Manitoba, Winnipeg, Canada

**Keywords:** ampicillin-resistant *Escherichia coli*, natural compound, metabolic regulation, pyruvate metabolism

## Abstract

Antimicrobial resistance has emerged as a major global public health threat, underscoring the urgent need for novel therapeutic strategies. In this study, we demonstrate that protocatechualdehyde (PA), a natural compound derived from *Salvia miltiorrhiza*, exhibits potent and time-dependent bactericidal activity against ampicillin-resistant *Escherichia coli*. PA was also effective against AmpC β-lactamase-expressing strains and clinically isolated multidrug-resistant strains of *E. coli*, *Pseudomonas aeruginosa*, and *Klebsiella pneumoniae*, notably by exerting a combined effect with aminoglycoside antibiotics to overcome resistance. Mechanistically, integrated metabolomic and functional analyses reveal that PA induces a profound metabolic reprogramming in ampicillin-resistant *Escherichia. coli*, characterized by the hyperactivation of central carbon metabolism, with pyruvate catabolism serving as a critical hub. This forced metabolic flux leads to a surge in intracellular ATP and NADH, ultimately driving an overload of the electron transport chain and a lethal burst of reactive oxygen species (ROS). Genetic and chemical inhibition of the pyruvate dehydrogenase complex attenuates both ROS production and the bactericidal effect, confirming the causal link between metabolic disruption and bacterial death. PA treatment markedly improved survival and reduced bacterial burden in a murine systemic infection model, suggesting its therapeutic potential for infections. These findings provide a foundational rationale for developing PA-based therapeutics or derivatives to combat multidrug-resistant Gram-negative infections, particularly in combination with aminoglycoside antibiotics.

**IMPORTANCE:**

The rising prevalence of multidrug-resistant Gram-negative pathogens is limiting treatment options. This study identifies the natural compound PA as an effective bactericidal agent against ampicillin-resistant and clinically relevant multi-drug-resistant (MDR) *Escherichia coli* and other Gram-negative species. Importantly, we elucidate a previously unreported mechanism whereby PA hijacks bacterial central metabolism, specifically pyruvate metabolism, leading to metabolic overactivation, accumulation of NADH and ATP, and ultimately lethal reactive oxygen species (ROS) production. Furthermore, under the combined effect of PA and aminoglycoside antibiotics, their minimum inhibitory concentrations are reduced against resistant strains. These findings support the therapeutic potential of PA as either a standalone or adjunctive treatment for drug-resistant infections. This work emphasizes the value of targeting bacterial metabolism as a viable strategy to combat antimicrobial resistance.

## INTRODUCTION

Pathogenic *Escherichia coli* (*E. coli*) causes various diseases, such as urinary tract infections, intestinal diarrhea, and neonatal meningitis, and its serious drug resistance leads to difficulties in clinical treatment (1, 2). The rising prevalence of multidrug-resistant *E. coli* strains has become a significant clinical concern, complicating treatment protocols and posing substantial public health challenges worldwide (3–5). Notably, resistance to ampicillin (AMP), one of the most historically used β-lactam antibiotics, has been increasingly reported in both community and healthcare settings. Epidemiological surveillance across various regions and sources consistently highlights ampicillin resistance as one of the most common phenotypic resistances observed in *E. coli* isolates, underscoring its role as a key indicator of broader resistance trends (6, 7). Thus, it is critical to develop novel antibacterial strategies to counteract the escalating challenge of bacterial resistance. Therefore, seeking new drugs that can overcome or bypass existing drug resistance mechanisms is an important strategy.

Nowadays, first-generation antibacterial agents, such as penicillin, have seen their clinical use dramatically decline due to the high prevalence of bacterial resistance (8). While second-generation agents like β-lactam antibiotics remain central to the antimicrobial arsenal, resistance to them is also widespread (9). In fact, to preserve their efficacy, drugs like AMP are rarely prescribed alone but are instead combined with β-lactamase inhibitors. Many Gram-negative bacteria have evolved complex resistance mechanisms to counteract the effects of β-lactam antibiotics, such as *Escherichia coli*, *Pseudomonas aeruginosa, Acinetobacter baumannii, and Klebsiella pneumoniae* (10). Consequently, research efforts are expanding in parallel to explore complementary strategies, including investigating bioactive compounds from traditional medicines. Recent evidence suggests that targeting bacterial metabolism can restore susceptibility to conventional antibiotics (11). For example, sodium formate was shown to enhance the bactericidal activity of micronomicin by promoting the metabolism of pyruvic acid to formic acid in multi-drug resistant (MDR) carbapenem-resistant *E. coli* (12). Sodium pyruvate disrupts cellular redox balances, improving gentamicin penetration in *Vibrio alginolyticus* (13). Exogenous alanine sensitizes *Vibrio alginolyticus* to gentamicin via accelerated nitric oxide metabolism (14). This suggests that certain metabolic modulators may overcome drug resistance.

Past reports showed that a natural compound, protocatechualdehyde (PA), possessed various biological activities, including anti-bacterial (15), anti-inflammatory (16), atherosclerosis reduction (17), antioxidant (18), and prevention of pulmonary fibrosis (19). Recent studies have shown that PA has a bactericidal effect on various non-drug-resistant bacteria, such as *Ralstonia solanacearum*, *Vibrio parahaemolyticus* ATCC17802, *Yersinia enterocolitica* BNCC108930, and *Listeria monocytogenes* ATCC19114 (20–23). The bactericidal effect of PA on drug-resistant bacteria has only been reported in methicillin-resistant *Staphylococcus aureus*, and its efficacy relies on the generation of reactive oxygen species (ROS) (24). However, the efficacy of PA against a broader range of drug-resistant Gram-negative pathogens and the mechanism by which it triggers ROS production are not well defined. Therefore, this study investigates the bactericidal activity and metabolic effects of PA on drug-resistant Gram-negative bacteria, with a focus on ampicillin-resistant strains presenting serious clinical resistance.

In this study, we first investigated the bactericidal effect of PA on ampicillin-resistant *Escherichia coli* (ECO-R_AMP_) and other Gram-negative drug-resistant bacteria. Given its efficacy, we then employed GC-MS-based metabolomics to elucidate the metabolic perturbations induced by PA, aiming to uncover the specific pathways responsible for enhanced ROS production and bacterial death. Key mechanisms were further validated using ROS scavengers, metabolic enzyme inhibitors, and quantification of intracellular PA accumulation. Finally, the therapeutic efficacy of PA was assessed in a mouse systemic infection model, examining its ability to reduce bacterial burden in major organs and improve host survival.

## RESULTS

### PA rapidly eradicates high-burden ampicillin-resistant and multidrug-resistant Gram-negative pathogens

**Fig 1:**
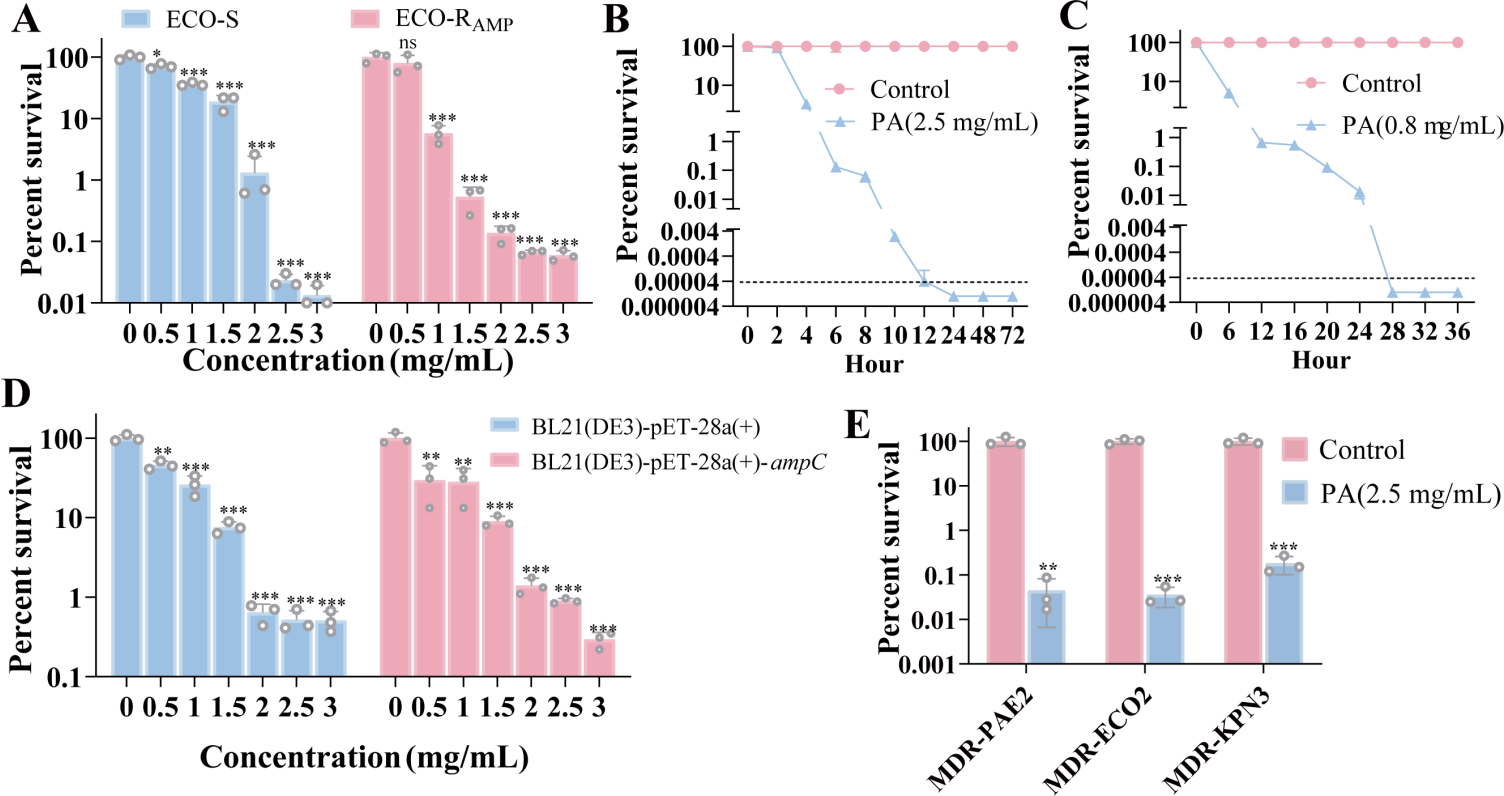
Effect of PA on the survival rate of ECO-R_AMP_. (**A**) Percent survival of ECO-S and ECO-R_AMP_ in the indicated concentration of PA, for 6 h. (**B**) Percent survival of ECO-R_AMP_ in the indicated incubation periods, exposure to 2.5 mg/mL PA, the dotted line indicates the limit of detection with 200 CFU/mL. (**C**) Percent survival of ECO-R_AMP_ in the indicated incubation periods, exposure to PA of 1× MIC (0.8 mg/mL), the dotted line indicates the limit of detection with 200 CFU/mL. (**D**) Percent survival of BL21(DE3)-pET-28a(+)-*ampC* and BL21(DE3)-pET-28a(+) exposure to 2.5 mg/mL PA, for 6 h. (**E**) Percent survival of multidrug-resistant *Pseudomonas aeruginosa* 2 (MDR-PAE2), *Escherichia coli* 2 (MDR-ECO2), and *Klebsiella pneumoniae* 3 (MDR-KPN3) exposure to 2.5 mg/mL PA, for 6 h. (*P < 0.05, **P < 0.01, ***P < 0.001, ns = not significant).

To model severe infections involving high bacterial loads (approximately 10^8^ colony-forming units [CFU]/mL) of resistant pathogens and to assess the rapid-response effectiveness of PA, we first conducted a comprehensive phenotypic analysis of AMP-resistant bacteria (ECO-R_AMP_) obtained from susceptible *Escherichia coli* K12 BW25113 (ECO-S) under AMP passage. The results revealed an elevated AMP minimum inhibitory concentration (MIC) of 0.064 mg/mL, a growth profile consistent with resistance, and mutations in the *rpoD* and *cpxA* genes, which may be associated with AMP resistance (25, 26), thereby confirming the acquisition of a resistant phenotype (Fig. S1). Based on previously reported literature (27), we performed a survival rate assay for ECO-S and ECO-R_AMP_ at a PA concentration gradient of 0, 0.5, 1, 1.5, 2, 2.5, and 3 mg/mL for 6 h. After 6 h of PA exposure, the survival rates of ECO-S and ECO-R_AMP_ decreased from 71.017% to 0.013% and from 79.747% to 0.059%, respectively, as the concentration increased from 0.5 to 3 mg/mL (Fig. 1A). Notably, the bactericidal efficiency of PA does not depend on whether the strain acquires AMP resistance and stabilizes at 2.5 mg/mL, close to the best bactericidal efficiency. Therefore, this concentration was selected to validate PA’s bactericidal efficiency on ECO-R_AMP_ over time and against other drug-resistant bacteria. With prolonged exposure to 2.5 or 0.8 mg/mL PA, the survival rate declined to 0% at 12 or 26 h, respectively, and no regrowth was observed after continued cultivation for more than 36 h (Fig. 1B and C). Furthermore, we tested the engineered AmpC β-lactamase-producing strain BL21(DE3)-pET-28a(+)-*ampC* and its empty-vector control BL21(DE3)-pET-28a(+). PA retained potent antibacterial activity against the AmpC-expressing strain (Fig. 1D). Additionally, we evaluated the activity of PA against clinically isolated multidrug-resistant strains whose MICs were listed in Table S1. After a 6 h treatment with 2.5 mg/mL PA, the clinical strains MDR-PAE2*,* MDR-ECO2, and MDR-KPN3 also halved survival rates, reaching 0.044%, 0.035%, and 0.18%, respectively (Fig. 1E). We further determined MICs and fractional inhibitory concentration (FIC) indices for PA combined with four common types of antibiotics, e.g., carbapenems (meropenem), quinolones (balofloxacin), cephalosporins (cefuroxime), and aminoglycosides (amikacin and micronomicin). PA combined with aminoglycosides (amikacin and micronomicin) lowered its own MIC by 64-fold and halved the antibiotic MICs in ECO-R_AMP_. In contrast, combining PA with balofloxacin or micronomicin primarily reduced the MICs of the latter by 1,024-fold and 256-fold, respectively, while modestly lowering the PA MIC twofold in MDR-ECO2 (Table S2), indicating an additive effect. This indicates that PA has a broad-spectrum bactericidal effect in a high-burden setting, and it helps to broaden the application of existing antibiotics. Thus, it is necessary to explore its action mechanism.

### PA can enter the cells and exert its effects

**Fig 2:**
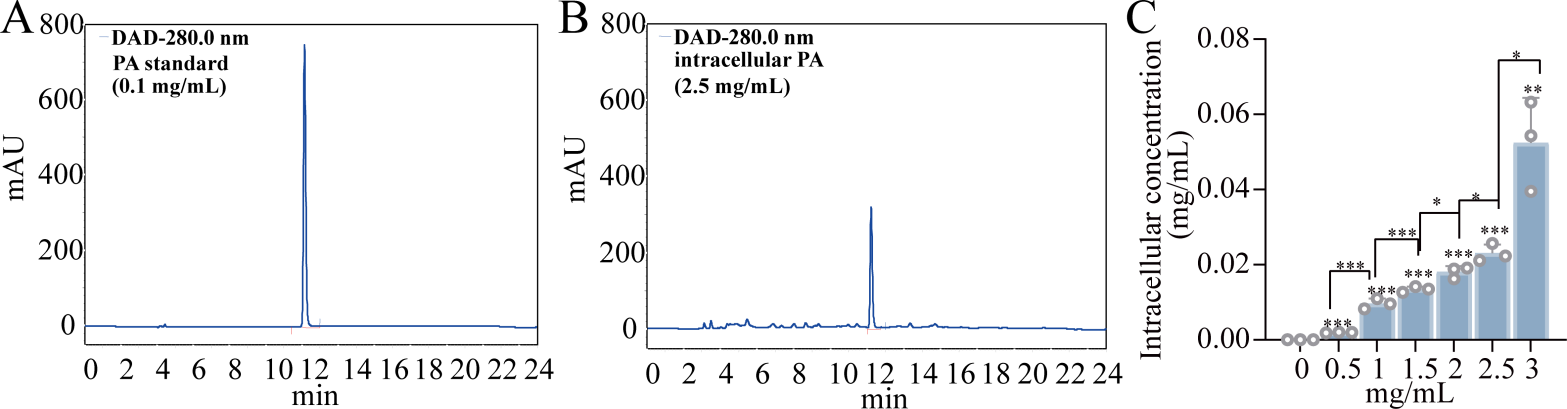
Detection of intracellular PA content by HPLC method. (**A**) HPLC chromatogram of standard 0.1 mg/mL PA. (**B**) HPLC chromatogram of intracellular PA (exogenous addition of 2.5 mg/mL PA). (**C**) Intracellular PA content in ECO-R_AMP_ in the indicated concentration of exogenous PA addition. (*P < 0.05, **P < 0.01, ***P < 0.001).

To determine whether PA can enter bacterial cells to exert its bactericidal effect, the intracellular PA concentration after treatment with a concentration gradient of PA was measured using high-performance liquid chromatography (HPLC) (28). Firstly, the peak area of 0.1 mg/mL PA standards was detected as shown in Fig. 2A. When the usage concentration was 2.5 mg/mL, the peak plot of intracellular PA content was shown in Fig. 2B. The determination of intracellular PA content was shown in Fig. 2C by calculating based on the standard curve in Fig. S2. It can be known that when PA was applied at concentrations of 0.5, 1.0, 1.5, 2.0, 2.5, and 3.0 mg/mL, the intracellular accumulation accounted for 0.39%, 0.96%, 0.89%, 0.90%, 0.92%, and 1.75% of the total applied amount, respectively. This result indicated that PA enters bacterial cells to exert its effect as the antibiotics that have been reported (12, 29), subsequently activating pyruvate metabolism and promoting the accumulation of ROS, mediating bactericidal effects.

### Global metabolomic reprogramming induced by PA in ECO-R_AMP_

**Fig 3:**
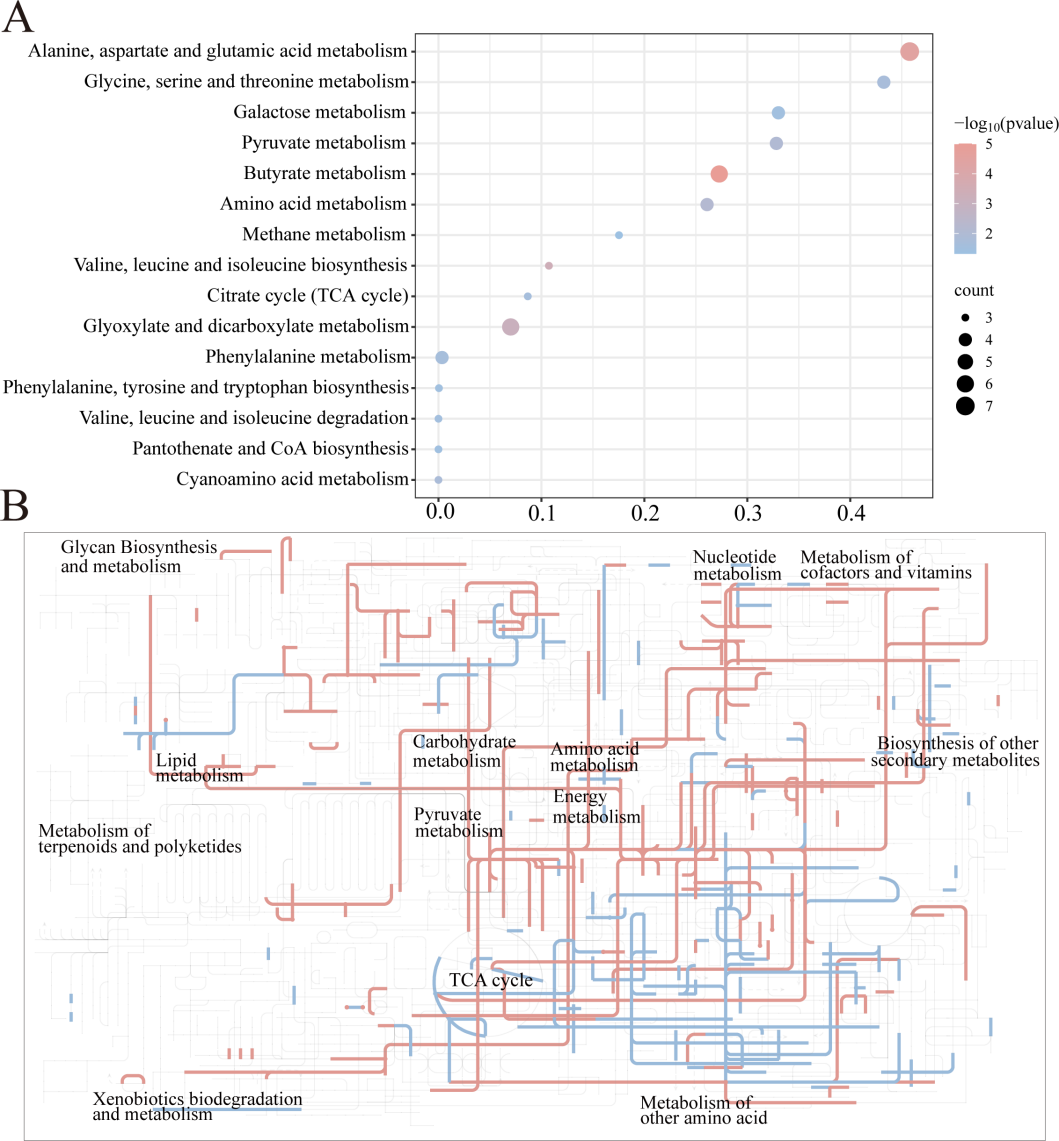
Differential metabolic pathway enrichment. (**A**) Differential metabolic pathways in ECO-R_AMP_ with PA compared with the control group. (**B**) iPath analysis (“red” means upregulation; “blue” means downregulation).

To investigate the metabolic mechanisms underlying the killing effect of PA, we employed a GC-MS-based metabolomics approach to compare the metabolic changes in ECO-R_AMP_ with or without 2.5 mg/mL PA (control). Quality controls confirmed high reproducibility with a correlation of 0.994 between technical replicates and clear separation between groups (Fig. 3). Differential analysis of metabolic abundances between the PA group and control group identified a total of 59 differential metabolites (Fig. S4A). Their classification is shown in Fig. S4B and spans multiple functional classes. The Z-score plot of differential metabolites indicated that the Z-values in the PA group ranged from −10.78 to 258.45 (Fig. S4C). These results demonstrate that PA induces a marked shift in the metabolome of ECO-R_AMP_, with widespread changes in metabolite levels.

### PA activates central carbon metabolic pathways with emphasis on pyruvate metabolism

To analyze the metabolic alterations in bacteria following PA treatment, various metabolic pathways were identified using MetaboAnalyst 6.0 (http://www.metaboanalyst.ca). A total of 15 metabolic pathways were determined. Among them, the top five metabolic pathways were alanine, aspartic acid and glutamic acid metabolism, glycine, serine, and threonine metabolism, galactose metabolism, pyruvate metabolism, and butyrate metabolism (Fig. 3A). While these alterations may reflect broader metabolic stress or adaptation in ECO-R_AMP_ under PA treatment, further investigation is needed to determine whether they contribute to PA’s bactericidal activity. To visualize these perturbed pathways within the global metabolic network and understand their interconnections, we performed an integrative analysis using iPath 3.0. The iPath mapping revealed that PA treatment induced extensive remodeling of central carbon and energy metabolism in ECO-R_AMP_ (Fig. 3B). Notably, pyruvate metabolism served as a critical hub, exhibiting direct and extensive connections to several other significantly altered pathways, including the alanine, aspartate, glutamate metabolism, and glycine, serine, and threonine metabolism. This network-level visualization underscored that PA-triggered metabolic changes were not isolated but centered around key junctions of energy and precursor supply. The prominent position of pyruvate metabolism in this interaction network, coupled with its high enrichment score, provided a compelling rationale for the hypothesis that activation of pyruvate flux and its linked pathways is central to PA’s mechanism.

### Identification of key metabolic biomarkers linked to PA-induced bacterial killing

**Fig 4:**
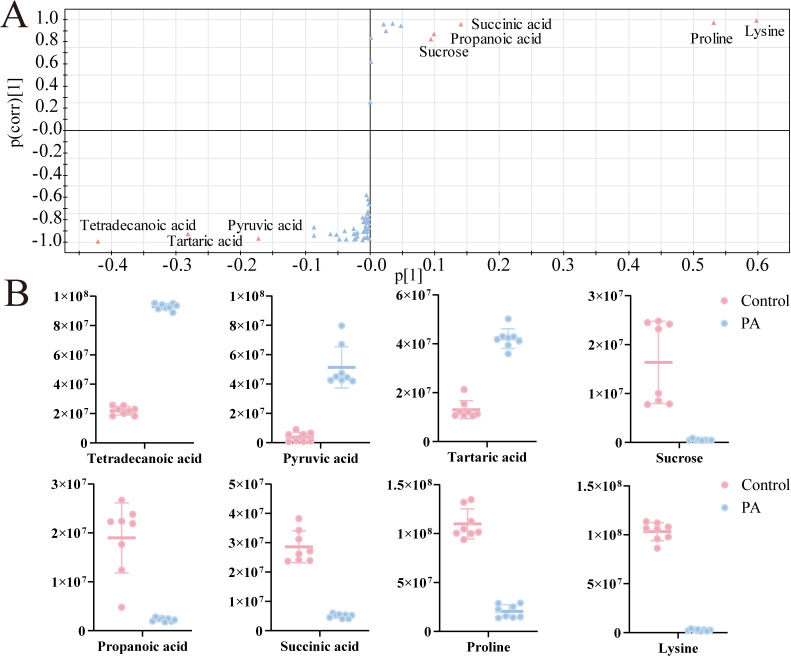
Differential metabolic target analysis. (**A**) S-plot generated from orthogonal partial least squares discriminant analysis of metabolic profile data for ECO-R_AMP_ with PA compared with control group. In the plot, metabolites are represented by triangles, with potential biomarkers highlighted in red, having absolute values of covariance *P* and correlation *P* (corr) greater than or equal to 0.05 and 0.5, respectively. (**B**) The scatter plot of metabolites from panel **A**.

To identify biomarkers of bacterial metabolism affected by PA, an S-plot analysis was conducted using OPLS-DA. In the plot, metabolites were represented by triangles, with potential biomarkers highlighted in red, having absolute values of covariance p and correlation p (corr) greater than or equal to 0.05 and 0.5, respectively. Through this analysis, eight biomarkers were identified (Fig. 4A). The scatter plot revealed that the abundance levels of tetradecanoic acid, pyruvic acid, and tartaric acid were upregulated, while those of sucrose, propionic acid, succinic acid, proline, and lysine were downregulated in the PA group (Fig. 4B). Among them, it is found that the top five pathways in Fig. 3A contain three biomarkers (pyruvic acid, succinic acid, and sucrose), both pyruvic acid and succinic acid served as key metabolic biomarkers, connecting pyruvate metabolism and the tricarboxylic acid cycle.

### PA exerts bactericidal effects in ECO-R_AMP_ through a ROS-dependent mechanism

**Fig 5:**
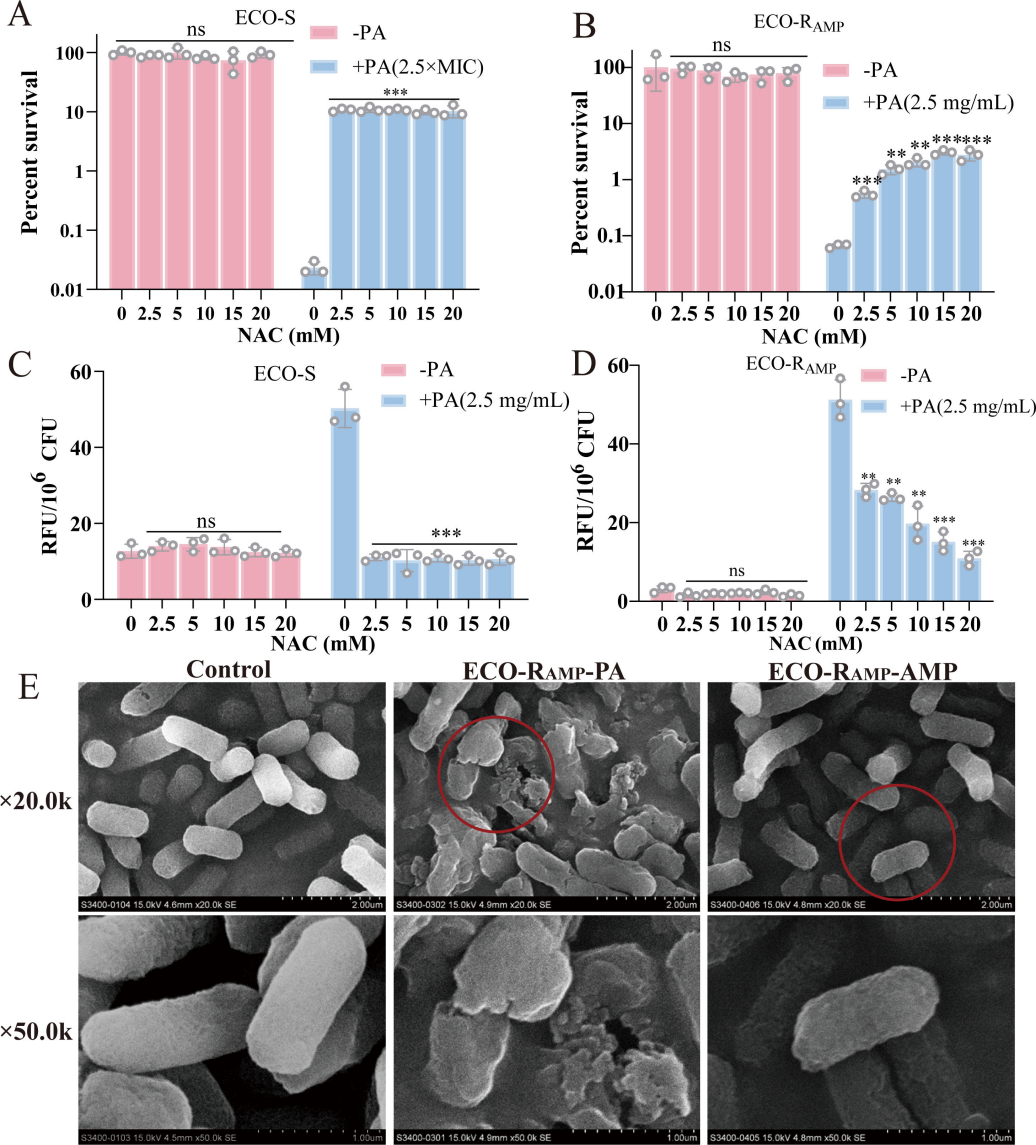
The role of ROS on PA’s bactericidal efficacy. (**A**) Percent survival of ECO-S in the indicated concentration of NAC with or without 2.5 mg/mL PA, for 6 h. (**B**) Percent survival of ECO-R_AMP_ in the indicated concentration of NAC with or without 2.5 mg/mL PA, for 6 h. (**C**) ROS level analysis in ECO-S with concentration gradient of NAC with or without 2.5 mg/mL PA by DCFH-DA method. (**D**) ROS level analysis in ECO-R_AMP_ with concentration gradient of NAC with or without 2.5 mg/mL PA by DCFH-DA method. (**E**) Observation of the appearance of ECO-R_AMP_ without PA (control), with 2.5 mg/mL PA, or 2.5 mg/mL AMP, respectively. The 50.0k images of ECO-R_AMP_-PA and ECO-R_AMP_-AMP were higher-magnification views of the areas outlined by the red circles in their corresponding 20.0k images, respectively. (**P < 0.01, ***P < 0.001, ns = not significant).

To determine whether the bactericidal effect of PA is dependent on ROS accumulation as previously reported (27), we conducted a series of validation experiments using an ROS inhibitor, quantitative ROS measurement, and morphological observations. The survival rate of ECO-S and ECO-R_AMP_ was assessed with PA under the action of ROS inhibitor N-acetylcysteine (NAC) with and without PA. The bactericidal efficacy of PA was decreased by NAC (Fig. 5A and B). The PA-induced elevation of ROS levels in both ECO-S and ECO-R_AMP_ was similarly diminished (Fig. 5C and D). Among them, the untreated control showed 100% survival and a baseline ROS level of 3.07 RFU in ECO-R_AMP_. Following treatment with 2.5 mg/L PA (0 mM NAC), bacterial survival dropped to 0.07%, while ROS levels surged to 51.23 RFU. Co-treatment with increasing concentrations of NAC (2.5, 5, 10, 15, and 20 mM) led to a dose-dependent recovery in survival to 0.48%, 1.46%, 1.98%, 3.00%, and 2.69%, respectively, and a concomitant decrease in ROS levels to 28.27, 26.41, 19.7, 15.09, and 10.88 RFU, respectively. This inverse correlation indicates that the partial rescue of bacterial survival was directly linked to the scavenging of PA-induced ROS. Notably, at the 20 mM NAC concentration, survival increased approximately 42.9-fold, whereas ROS levels were reduced by about 3.54-fold. This indicated that NAC achieved only a partial mitigation of the oxidative stress. It remains clear that PA indeed relies on ROS to exert its bactericidal effect. Scanning electron microscopy (SEM) results showed that the morphology of ECO-R_AMP_ cells without any treatment was normal (control). Under the treatment of PA, the bacteria were seriously damaged, while AMP treatment did not cause significant damage (Fig. 5E). The results show that PA can cause bacterial rupture and death by promoting the production of ROS. Based on the centrality of pyruvate and the known link between enhanced central carbon metabolism, electron transport chain activity, and ROS generation (30, 31), we formulated the specific hypothesis that PA-induced pyruvate metabolism activation drives lethal ROS production. However, whether the production of ROS is related to the disturbance of pyruvate metabolism still needs further study.

### PA triggers hyperactivation of pyruvate metabolism, leading to ROS burst and bacterial death

**Fig 6:**
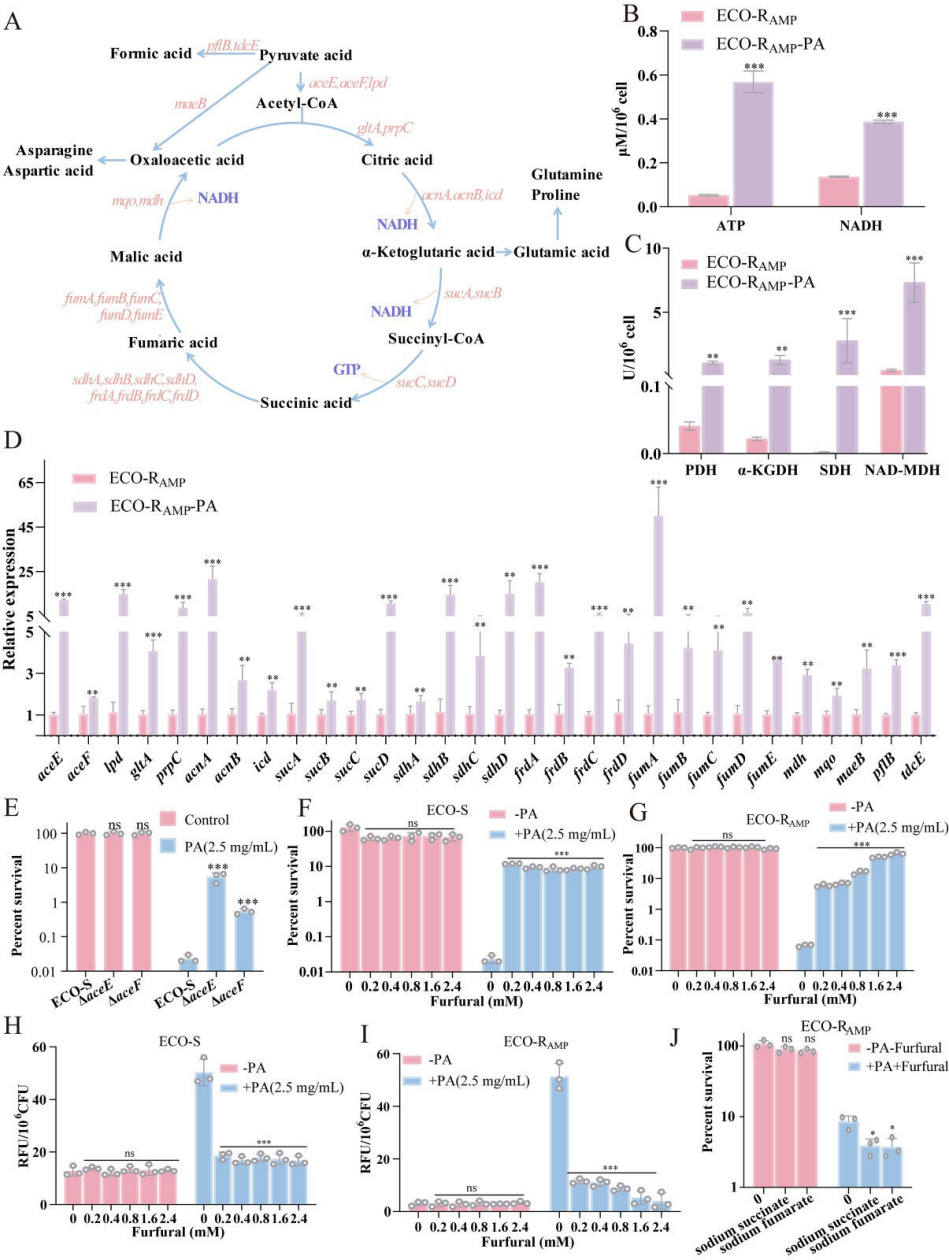
The effect of PA on the pyruvate metabolic pathway. (**A**) Diagram of pyruvate metabolism pathway in *Escherichia coli*. (**B**) Determination of NADH and ATP levels in ECO-R_AMP_ with or without 2.5 mg/mL PA. (**C**) Determination of enzyme activity levels in ECO-R_AMP_ with or without 2.5 mg/mL PA. (**D**) The changes in the mRNA expression levels of genes involved in the pyruvate metabolism pathway ECO-R_AMP_ and ECO-R_AMP_-PA (treated with 2.5 mg/mL PA for 6 h). (**E**) Percent survival of ECO-S and mutant strain *ΔaceE and ΔaceE* with or without 2.5 mg/mL PA, for 6 h. (**F**) Percent survival of ECO-S in the indicated concentration of furfural with or without 2.5 mg/mL PA, for 6 h. (**G**) Percent survival of ECO-R_AMP_ in the indicated concentration of furfural with or without 2.5 mg/mL PA, for 6 h. (**H**) ROS level in ECO-S with a concentration gradient of furfural with or without 2.5 mg/mL PA. (**I**) ROS level in ECO-R_AMP_ with a concentration gradient of furfural with or without 2.5 mg/mL PA. (**J**) Percent survival of ECO-R_AMP_ in the indicated concentration of sodium succinate (10 mM) and sodium fumarate (10 mM), with or without 2.5 mg/mL PA and 0.2 mM furfural, for 6 h. (*P < 0.05, **P < 0.01, ***P < 0.001, ns = not significant).

To verify that PA promotes ROS production by hyperactivating pyruvate metabolism (Fig. 6A, schematic), we analyzed this central pathway. At first, we detected the activity levels of ATP, NADH, related enzymes, and gene expression levels of bacteria with or without 2.5 mg/mL PA. It was found that the ATP content and NADH content of ECO-R_AMP_ increased significantly upon PA treatment (Fig. 6B). In addition, PA promotes the activity of enzymes pyruvate dehydrogenase (PDH), α-ketoglutarate dehydrogenase (α-KGDH), succinate dehydrogenase (SDH), and malate dehydrogenase (MDH) associated with pyruvate metabolism (Fig. 6C). Among them, the activity of SDH and MDH was very strongly upregulated, which leads to the downregulation of succinic acid and is consistent with the results of the previous metabolome analysis (Fig. 3B). To further verify that PA treatment can influence pyruvate metabolism, we measured the expression levels of genes involved in pyruvate metabolism. Among the 30 genes detected, after PA treatment, the mRNA expression levels of ECO-R_AMP_ increased significantly (Fig. 6D). The genes *aceE* and *aceF* encode core catalytic subunits of the pyruvate dehydrogenase complex. We tested the survival rate of gene mutant strains Δ*aceE* and Δ*aceF* from ECO-S under the action of PA. It showed that the survival rate of Δ*aceE* and Δ*aceF* was significantly higher than wild-type ECO-S (Fig. 6E). Then, an inhibitor furfural of PDH was used to analyze survival rates (32). In ECO-S, 0.2 mM furfural can significantly inhibit the bactericidal effect of PA (Fig. 6F). While furfural was added alone, there was no reduction in ECO-S and ECO-R_AMP_ bacterial survival rate; however, furfural combined with PA increased the survival rate to 64.46% (Fig. 6G). Moreover, the results of ROS level after the addition of furfural and PA showed that furfural effectively inhibited the level of ROS in ECO-S and ECO-R_AMP_ (Fig. 6H and I). Since succinic acid and fumaric acid are important products of the TCA cycle. These two substances were used in combination with PA and furfural, and the inhibitory effect of furfural on PA is reduced (Fig. 6J). These data suggest that pyruvic acid metabolism, particularly its flux into the TCA cycle, may contribute to the action of PA.

### PA enhances survival and reduces bacterial load in murine systemic infection models

**Fig 7:**
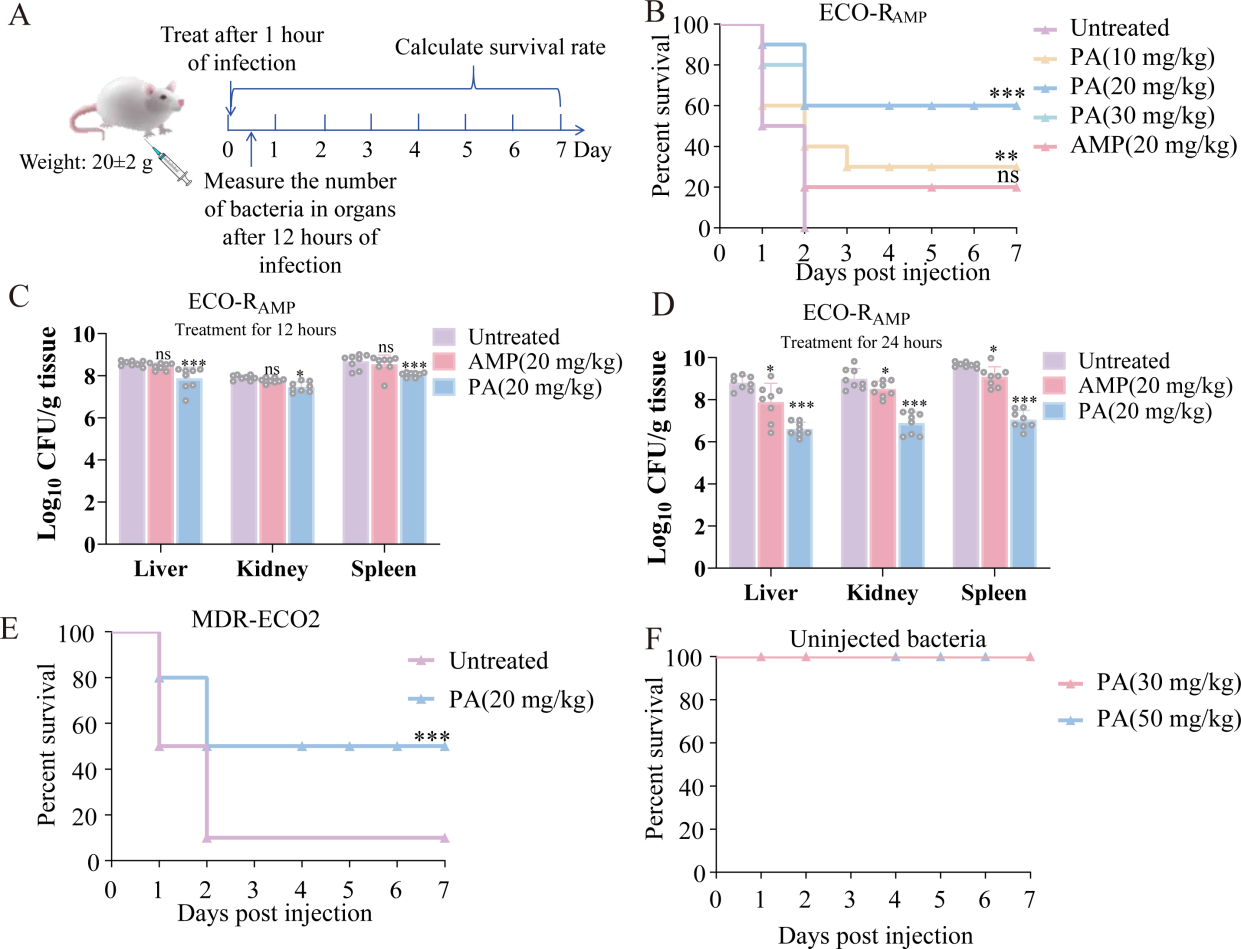
The effect of PA on the survival ability of mice. (**A**) Mouse infection model. (**B**) Survival rate of infected mice treated with PA, 10 mice in each group. (**C**) and (**D**) Determination of organ bacterial count in infected mice before and after treatment for 12 h (**C**) or 24 h (**D**) with PA, 8 mice in each group. (**E**) Survival rate of mice infected with MDR-ECO2 before and after treatment with PA, 10 mice in each group. (**F**) The survival rate of mice under the sole action of PA, 10 mice in each group. The body weight of all the tested mice was 20 ± 2 g. (*P < 0.05, **P < 0.01, ***P < 0.001, ns = not significant).

To investigate the anti-infective efficacy of PA *in vivo*, a mice infection model was established with subsequent drug administration (Fig. 7A). Compared to the group without PA (untreated group), preliminary dose-ranging experiments revealed that PA administration at 5 and 10 mg/kg increased survival rates by 10% and 30%, respectively (Fig. S5A). This inspired us to further increase the concentration of PA to determine whether PA could exert a better protective effect against infection in mice. Therefore, we selected 10 mg/kg as the lowest dose and conducted formal experiments with 10 mice in each group. When the dose concentration was 10 mg/kg, PA increased the survival rate of mice by 30%. At concentrations of 20 and 30 mg/kg, the survival rate was improved by 60% (Fig. 7B). The mice that were successfully treated by PA were able to continue eating and moving normally. On the contrary, the mice that were not successfully treated would exhibit symptoms, such as an inability to eat and reduced spontaneous activities. This dose significantly reduced bacterial burdens in the liver, kidney, and spleen compared with untreated mice (Fig. 7C and D). Specifically, after 24 h of treatment with AMP or PA, the residual bacterial loads in the liver were reduced to 34.67% or 0.65%, in the kidney to 24.59% or 0.74%, and in the spleen to 45.84% or 0.36%, respectively (Fig. 7D). In mice infected with ECO-R_AMP_ at inoculate of 3.36 × 10^15^ CFU, 2.02 × 10^15^ CFU, and 1.68 × 10^15^ CFU without treatment, the survival rates after 24 h were 0%, 40%, and 60%, respectively (Fig. S5B). These data indicated that a reduction in bacterial load by approximately 50% from 3.36 × 10^15^ to 1.68 × 10^15^ CFU was associated with a markedly higher survival rate. It indicated that PA treatment successfully lowered the pathogen load to a level that could be effectively managed by the host immune system, thereby converting a lethal infection into a non-lethal one. In addition, PA also exhibited efficacy against MDR-ECO2, boosting survival by 40% in infected mice (Fig. 7E). Notably, no abnormal activities, such as death, were observed in mice when a high dose of 50 mg/kg of PA was injected alone (Fig. 7F). Moreover, cytotoxicity determination showed that the IC₅₀ value of PA on Verda Reno cells was greater than 1 mg/mL (Fig. S6). Together with its demonstrated efficacy in enhancing host defense against both artificially passaged and clinically isolated drug-resistant bacterial infections, these findings were meaningful for further exploring the application of PA or its derivatives as therapeutic substances or disinfectants for daily chemical products.

## DISCUSSION

Antimicrobial resistance has become a critical and escalating challenge for global public health. According to the 2023 report from the China Antimicrobial Resistance Surveillance System, *E. coli* has remained the most frequently isolated clinical pathogen for 18 consecutive years. The antimicrobial resistance rate of 80,609 strains of *E. coli* showed that antibiotics with a resistance rate greater than 50% were ampicillin, piperacillin, ciprofloxacin, cefazolin, levofloxacin, cefuroxime, cotrimoxazole, and ceftriaxone in order from high to low. Among them, ampicillin topped the list with a resistance rate of 84.1% (33). The escalating prevalence of β-lactamase-resistant, such as ampicillin-resistant *E. coli,* underscores the urgent need for novel therapeutic agents and strategies that aim to operate through mechanisms distinct from existing antibiotics to overcome or bypass common resistance pathways (34–37). Natural products are valuable resources for new drug discovery. PA, the active ingredient from *Salvia miltiorrhiza,* has been reported to kill a variety of sensitive bacteria and drug-resistant *Staphylococcus aureus* through a ROS-dependent mechanism (21, 27, 38). However, its efficacy against drug-resistant Gram-negative bacteria remained unclear.

In this study, we analyzed the phenotype of resistant bacteria ECO-R_AMP_ obtained from susceptible bacteria ECO-S. It showed ECO-R_AMP_ exhibited an eightfold increase in ampicillin MIC relative to the ECO-S and demonstrated a reduced growth rate, consistent with the fitness cost often associated with resistance acquisition. This reduced growth may stem from the metabolic burden of expressing resistance mechanisms, such as β-lactamase production, which consumes substantial energy and resources (16). This suggests that after bacteria develop drug resistance, their metabolic levels will change significantly. Whole-genome sequencing identified missense mutations in *rpoD* (25) (encoding RNA polymerase sigma factor) and *cpxA* (26) (encoding a sensor histidine kinase), both of which have previously been linked to antibiotic resistance, including ampicillin resistance in *E. coli*.

In the antibacterial analysis, we demonstrated that PA exhibits potent bactericidal activity against a panel of sensitive bacteria, ECO-S, and drug-resistant Gram-negative bacteria, including laboratory-generated ampicillin-resistant ECO-R_AMP_ and clinically isolated multidrug-resistant strains of MDR-ECO2, MDR-PAE2, and MDR-KPN3, highlighting its broad-spectrum potential. Notably, PA’s efficacy was not compromised by the presence of the AmpC β-lactamase, a common resistance determinant, indicating its potential to circumvent typical β-lactam resistance mechanisms. Furthermore, PA acted as an effective adjuvant for aminoglycoside or quinolone antibiotics, significantly lowering their effective MICs. This combination aligns with emerging strategies where metabolic modulation potentiates conventional antibiotics (12–14). The difference was that PA can effectively eliminate ampicillin-resistant bacteria, indicating that their mechanism of action may be different. Ampicillin mainly inhibits penicillin-binding protein, which is involved in cell wall biosynthesis, and the mutation of this coding gene in drug-resistant bacteria can easily lead to drug resistance (39–41). Based on the mechanism of PA proposed for methicillin-resistant *Staphylococcus aureus* (27), we hypothesize that in *E. coli*, PA is able to avoid this target and is highly efficient through ROS-mediated sterilization as previously reported (27).

Given the multitude of pathways associated with ROS, we first employed metabolomics to identify those potentially linked to ROS generation. GC-MS-based metabolomics revealed that PA treatment induces significant reprogramming of central carbon metabolism in ECO-R_AMP_, with a marked activation of pyruvate metabolism and its connected pathways. To confirm the role of ROS in PA-induced killing, we measured intracellular ROS levels. PA treatment triggered a significant increase in ROS within ECO-R_AMP_. Importantly, the addition of the ROS scavenger NAC simultaneously abolished both the ROS burst and the bactericidal effect of PA. This parallel rescue by NAC supports the involvement of ROS in the killing mechanism. HPLC and SEM analyses confirmed that PA enters bacterial cells and causes morphological damage, leading to cell rupture and death. These results are consistent with the ROS-dependent killing mechanism reported for methicillin-resistant *Staphylococcus aureus* (27). Based on the centrality of pyruvate and the known link between enhanced central carbon metabolism, electron transport chain activity, and ROS generation (30, 31), we formulated the specific hypothesis that PA-induced pyruvate metabolism activation drives lethal ROS production.

**Fig 8:**
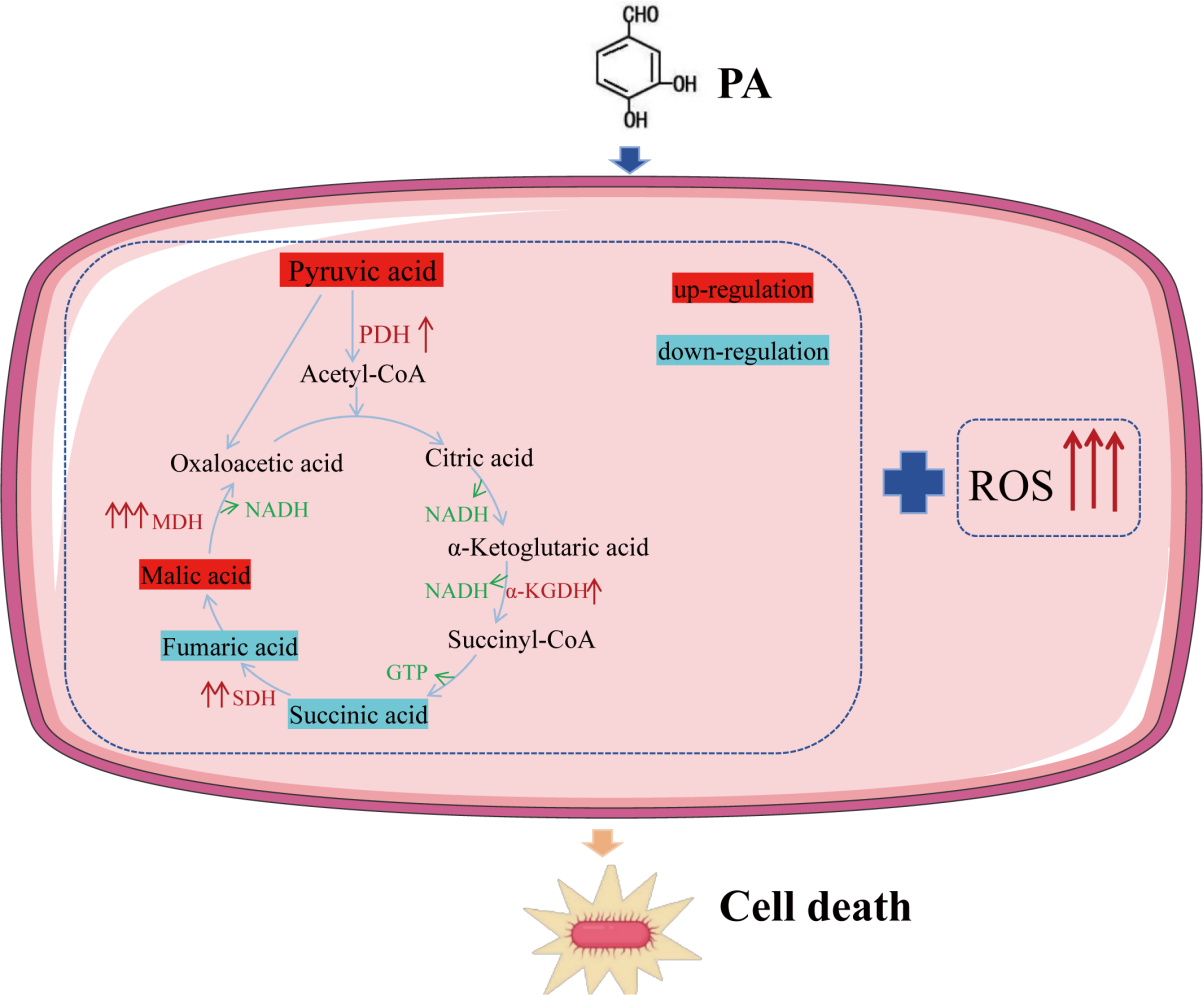
Mechanism of PA sterilization (“↑” means upregulation; “↓” means downregulation).

Importantly, we linked this hyperactive metabolic state to a lethal burst of ROS. Key metabolites, such as pyruvate, were altered, and this metabolic activation was accompanied by increased ATP and NADH production. Consistent with these findings, the activities of PDH, α-KGDH, SDH, and MDH were elevated, and the expression of 30 genes related to pyruvate and TCA metabolism was significantly enhanced. These results indicate that PA forces resistant bacteria into a hypermetabolic state, and we hypothesized that this metabolic rewiring drives ROS generation (32). Supporting this, we used furfural, a PDH inhibitor that reduces its activity by over 90%. Exogenous addition of furfural effectively suppressed both PA-induced ROS production and bactericidal activity. This finding was further supported by the increased survival of Δ*aceE* and Δ*aceF* mutant strains upon PA treatment. These results showed that PA could promote the ROS level by activating pyruvate metabolism (Fig. 8).

In a mouse model of systemic infection, PA treatment increased the survival rate of ECO-R_AMP_ infected mice by 60%, outperforming ampicillin at the same concentration by 40%. The decrease in the number of bacteria in the organs can indicate that the drug has a certain therapeutic effect (42). PA also significantly reduced bacterial loads in the liver, kidney, and spleen and demonstrated a 40% survival benefit in mice infected with MDR-ECO2. It is noteworthy that the reduction in bacterial load, though not complete sterilization, was sufficient to convert a lethal infection into a survivable one. Importantly, PA exhibited no acute lethality at doses up to 50 mg/kg and a high IC₅₀ in mammalian cells, which lays a foundation for its further application.

However, our study has limitations. The mechanistic insights discussed herein were primarily based on in *vitro* findings, while this work focused on elucidating the mechanism in ECO-R_AMP_. We note that future studies could explore the differential metabolic responses of ECO-S to PA, which may provide additional insights into its resistance adaptation. Subsequent *in vivo* studies are necessary to fully evaluate the therapeutic efficacy and safety of PA. Future studies should also further explore the antibacterial activity of PA derivatives against drug-resistant bacteria based on PA, particularly those that may allow for reduced dosages to meet clinical needs. Additionally, their potential for *in vitro* applications, such as disinfection in daily chemical products, should also be investigated.

### Conclusion

In summary, our study demonstrates that PA possesses potent efficacy against drug-resistant *E. coli* both *in vitro* and *in vivo*. We further elucidate that PA induces metabolic reprogramming in resistant bacteria, activating pyruvate metabolism and the TCA cycle, which leads to enhanced ROS production and bacterial death. Inhibition of pyruvate metabolism abrogates this ROS burst and rescues bacterial survival. This work broadens the application of PA to the combat against ampicillin-resistant *E. coli* and provides innovative insights into its mechanism of action through metabolic regulation. These findings underscore the potential of PA as a promising therapeutic agent or adjuvant against multidrug-resistant Gram-negative infections. Future studies will focus on identifying the precise bacterial target of PA and its derivatives or novel functions to further advance its therapeutic development.

## MATERIALS AND METHODS

### Strains and cultivation conditions

The bacterial strains employed in this study include artificial passage ampicillin-resistant *Escherichia coli* (ECO-R_AMP_), *sensitive Escherichia coli* K12 BW25113 (ECO-S), all of which were sourced from our laboratory collection. Clinical isolation of multi-drug-resistant *Escherichia coli* 2 (MDR-ECO2), *Pseudomonas aeruginosa* 2 (MDR-PAE2), and *Klebsiella pneumoniae* (MDR-KPN3) was provided by the Fourth Affiliated Hospital of Nanchang University. ECO-S and mutant bacteria Δ*aceE* and Δ*aceF* were from the Keio *Escherichia coli* Strain Preservation Center. BL21(DE3)-pET-28a(+)-*ampC* and its empty-vector control BL21(DE3)-pET-28a(+) stored at our laboratory. These bacteria were cultivated in 50 mL of Luria-Bertani (LB) broth (consisting of 1% bacto-peptone, 0.5% yeast extract, and 1% NaCl) at 37°C for 16–20 h. Following incubation, the cultures were centrifuged at 5,000 × *g* for 5 min, saline was suspended uniformly, and centrifuged under the same conditions to remove LB, repeat twice.

### Determination of MIC

According to Clinical and Laboratory Standards Institute guidelines, the MIC of ECO-R_AMP_ was measured by antimicrobial susceptibility testing. In brief, bacterial inoculation was performed using Mueller-Hinton base (MHB) medium (supplemented with 20 mg/L CaCl_2_ and 10 mg/L MgCl_2_) and shaken at 37°C and 200 rpm for 16–20 h. Subsequently, the bacterial suspension was transferred to fresh MHB at a 1:100 ratio and grown until the OD_600_ reached 0.5. Dilute the bacterial suspension to achieve a bacterial count of 5 × 10^6^ CFU/mL, and 10 μL of bacterial solution was added to each well in a 96-well plate, with each well containing 5 × 10^4^ CFU of bacteria. A twofold serial dilution method was employed to achieve PA (Sigma, 37520-25G)/antibiotic concentrations in MHB. The total volume of each well in the 96-well plate was 100 μL. After mixing the PA/antibiotics solutions with the bacterial suspensions, the plates were incubated at 37°C for 16 h. The minimum PA/antibiotics concentration that did not produce visible precipitates was the MIC. All antibiotic sources were listed in Table S3. Three biological replicates were conducted for each sample.

The efficacy of antibiotic combination therapy can be determined by calculating the FIC index. The formula for calculating the FIC index is FIC = MIC_antibiotic combination_/MIC_antibiotic alone_ + MIC_PA combination_/MIC_PA alone_. In the formula, MIC_antibiotic alone_ and MIC_PA alone_ represent the MIC values when the antibiotics and PA are used alone, while MIC_antibiotic combination_ and MIC_PA combination_ represent the MIC values when the antibiotics and PA are used in combination. Standard for the combined effect of drugs: when FIC ≤ 0.5, it shows a synergistic effect; when 0.5 < FIC ≤ 1, it shows an additive effect; when 1 < FIC ≤ 2, it shows an irrelevant effect; when FIC > 2, it shows an antagonistic effect (43).

### Detection of mutation sites in ECO-R_AMP_

Collect the bacteria (ECO-S and ECO-R_AMP_) in the logarithmic phase. Centrifuge 2 mL of the logarithmic phase bacterial solution at 14,000 × *g* and 4°C for 1 min, and discard the supernatant. The bacterial precipitate was rapidly frozen in liquid nitrogen and stored at −80°C for sequencing (Guangzhou Yiyunrui Biotechnology Co., Ltd.). As outlined in the referenced methodology, genomic variations between ECO-S and ECO-R_AMP_ strains were characterized using Illumina-based whole-genome resequencing (44). Following quality control of raw sequencing reads, strain-specific genetic variations were identified through comparative analysis. These variants were subsequently mapped and annotated using the well-annotated *E. coli* K-12 MG1655 reference genome by SnpEff (45). Three biological replicates were conducted for each sample.

### Measurement of bacterial growth curves

A single bacterial clone was inoculated into 5 mL of LB and shaken continuously (37°C, 200 rpm) for 16–24 h. The bacterial suspension was then transferred to fresh 50 mL LB at a 1:100 ratio, and the OD values of all cultures were adjusted to 0.012. The adjusted suspensions were dispensed into test tubes (5 mL each) and incubated in a shaker (37°C, 200 rpm). The absorbance at OD_600_ was measured every 2 h. Three biological replicates were conducted for each sample.

### Assessment of bacterial viability

A single bacterial clone was inoculated into 5 mL of LB and shaken continuously (37°C, 200 rpm) for 16–24 h. The bacterial suspension was transferred to fresh LB at a 1:1,000 ratio, and the OD values of all cultures were adjusted to the same level. The adjusted suspensions were dispensed into test tubes (5 mL each). Subsequently, 50 μL solutions at concentrations of 0, 1/6, 1/3, 1, 3, and 6× MIC of AMP were added to the tubes, which were then incubated in a shaker (37°C, 200 rpm) for 6 h. The absorbance at OD_600_ was measured. Three biological replicates were conducted for each sample.

### Bacterial survival rate assays

Bacterial survival rate assays were conducted as described previously (27). Well-grown bacterial cells were collected, washed two times, and resuspended in M9 medium containing 10 mM sodium acetate, 2 mM MgSO_4_, and 0.1 mM CaCl_2_ to a concentration of OD_600_ of 0.6 or 0.2 and diluted 100-fold. PA or other substances were added, and the mixture was incubated at 37°C and 200 rpm for 6 h (time gradient excepted). To determine CFU per mL, 100 μL samples were serially diluted 10-fold, and 5 μL aliquots were spotted onto LB agar plates. The plates were incubated at 37°C for 16 h. Only dilutions yielding 20–200 colonies were used to calculate CFU. The survival percentages of experimental groups were calculated using the untreated group as a control. Three biological replicates were conducted for each sample.

### Bacterial survival under time gradient of PA

In accordance with the methodology employed for bacterial survival rate experiments, a concentration of 2.5 mg/mL of PA was incorporated into the bacterial suspension during incubation. Following incubation periods, the samples were subjected to 10-fold serial dilutions using saline. After thorough mixing using a vortex mixer, 5 μL aliquots were dispensed onto LB agar square plates in a single spot. The plates were then incubated at 37°C for 10–16 h before enumeration. A control group, devoid of any added metabolites or antibiotics, was concurrently utilized to calculate the percentage survival rates of the experimental groups, respectively. Three biological replicates were conducted for each sample.

### Metabolic profiling

Each 1 mL of supernatant was transferred to a 1.5 mL QSP tube, and 0.1 mg/mL ribitol (internal standard) was added. The samples were dried using a vacuum centrifuge (Eppendorf, Germany) at 37°C to remove methanol completely. The dried samples were derivatized by adding 80 μL of 20 mg/mL methoxamine hydrochloride-pyridine solution, followed by incubation at 200 rpm at 37°C for 3 h with sonication assistance. Subsequently, 80 μL of N-methyl-N-(trimethylsilyl) trifluoroacetamide (MSTFA; Sigma-Aldrich, Cat. No. 394866) was added to each sample, followed by incubation at 37°C with shaking at 200 rpm for 30 min to complete derivatization. Finally, 120 μL of the derivatized supernatant was used for GC-MS analysis. Each sample was prepared with four biological replicates and two technical replicates, yielding a total of 16 data sets.

GC-MS analysis was performed on an Agilent 8890 gas chromatograph coupled with a 5975B mass spectrometer using an HP-5MS column (30 m, 250 µm i.d., 0.25 µm film thickness). The initial temperature was maintained at 70°C for 5 min, and the temperature was raised at 2°C/min to 270°C and maintained for 5 min. Each 1 μL sample was injected in splitless mode with an injector temperature of 270°C, interface temperature of 270°C, ion source temperature of 230°C, quadrupole temperature of 150°C, and ionization voltage of 70 eV. High-purity helium was used as the carrier gas at a flow rate of 1.0 mL/min. Full scan mode was employed, covering a mass range of 60–600 m/z.

Metabolites were identified using the National Institute of Standards and Technology (NIST) mass spectral database (version 2008) by comparing the electron ionization mass spectral fragments of detected metabolites with those of standard compounds stored in the database. Electron ionization mass spectrometry spectral information was analyzed using the NIST AMDIS (Automated Mass Spectral Deconvolution and Identification System) software. The obtained data were normalized based on total ion count, yielding standardized data, including metabolites, retention times, and peak areas, for further metabolomic analysis. IBM SPSS Statistics 19 was employed for significant difference analysis (*P* < 0.05). Cluster analysis and heatmap generation were performed using R software (R×64 3.6.1) for both the entire metabolome and differentially abundant metabolites. Principal component analysis and S-plot analysis were conducted using SIMCA-P+ 12.0 (version 12; Umetrics, Umea, Sweden), and metabolic pathway enrichment was achieved through MetaboAnalyst 4.0. Metabolic flux analysis was performed using iPath 3.0 (https://pathways.embl.de/). Final figures were prepared using GraphPad Prism 8.0 and Microsoft PowerPoint.

### Measurement of ROS content

According to the method of determining the survival rate, metabolites or ROS inhibitors were added to the bacterial solution, and ROS levels were measured after 6 h of incubation. Simultaneously measure the ROS levels of ECO-R_AMP_ and ECO-S without adding any substances. Mix 10^6^ CFU of bacteria with 10 μM ROS assay dye 2′,7′-dichlorofluorescein diacetate (DCFH-DA; Sigma, D6883) and incubate at 37°C for 30 min. The fluorescence intensity of ROS was measured by an enzyme label (Meigu Molecular Instruments, Spectramax M2) (F485 nm/F535 nm). Three biological replicates were conducted for each sample.

### Intracellular PA concentration measurement

Bacterial suspensions obtained after incubation (50 mL bacterial suspension) were processed following the survival rate assay method. After centrifugation at 5,000 × *g* for 5 min, the supernatant was discarded, and the pellet was resuspended in 10 mL of saline to adjust the OD_600_ to 1.0. Following centrifugation at 5,000 × *g* for 5 min, the supernatant was removed, and the pellet was transferred to a 1.5 mL QSP tube with 1 mL of saline. After centrifugation at 12,000 × *g* for 3 min, the supernatant was discarded, and 1 mL of pre-cooled (−80°C) methanol was added to the pellet. The bacterial cells were disrupted using ultrasound at 650 W × 35% power, with a 2 s pulse and 3 s interval for a total of 10 min. The supernatant was separated by centrifugation at 12,000 × *g* for 10 min at 4°C. Every 1 mL of supernatant was transferred to a 1.5 mL QSP centrifuge tube, and the supernatant was enriched with a balanced concentrator (Model: EYELA/CVE-3000) at a pressure of 24 hPa and a rotational speed of 6. The completely dried samples were reconstituted with 40 μL of methanol and transferred to the liner tube of a sample vial for HPLC analysis (model: Chromaster/Primade; manufacturer: Hitachi, Japan). The chromatographic separation was carried out on an Inertsil ODS-3 C_18_ column (250 mm × 4.6 mm, 5 μm). The detection wavelength of HPLC was 280 nm. The injection volume was 10 μL. The mobile phase consists of methanol (10%) and water (90%), and the flow rate is 0.75 mL/min. Three biological replicates were conducted for each sample.

### SEM analysis

The bacteria cultured overnight in LB medium were collected by centrifugation at 5,000 × *g* for 3 min, and the bacterial precipitates were washed with normal saline twice. Then, the bacteria were suspended in fresh LB medium, and the bacteria concentration was adjusted to an OD_600_ of 0.6, and divided into two groups. The experimental group was treated with PA and incubated in a shaker for 6 h, while the control group underwent identical processing without PA addition. The bacteria were suspended in fresh LB medium and adjusted to an OD_600_ of 0.6, then divided into two groups: this parallel treatment design ensures comparable conditions between groups while testing PA’s specific effects.

The incubated bacterial solution was adjusted for OD_600_ at 1.0, 20 mL bacterial solution was centrifuged, and 2.5% glutaraldehyde fixing solution was added to 40 times the volume and fixed at 4°C for more than 2 h. After fixation, centrifuge and discard the supernatant, rinse and precipitate with normal saline, and repeat three times. Gradient dehydration was carried out with 50%, 70%, 90%, and 100% ethanol successively, and the standing time of each dehydration was 10 min. After dehydration, 200 μL of 100% tert-butanol was added and suspended on the bacterial precipitate. After standing at 4°C for 30 min, the supernatant was centrifuged, and 200 μL 100% tert-butanol was added again to mix with the bacterial precipitate. Place in a freeze dryer and vacuum dry for 24 h before removing. After drying, the samples were gold-plated by ion sputtering and then observed using a scanning electron microscope (model: S-3400N; manufacturer: Hitachi) and photographed. Three biological replicates were conducted for each sample.

### Measurement of ATP content

According to the experimental method of survival rate, 2.5 mg/mL PA was added or not. After incubation for 6 h, the bacterial solution concentration was adjusted to 440 CFU/μL. Then, 50 μL of the above bacterial solution was taken into the 96-well plate. The linear value of ATPlite was immediately determined using a multi-functional enzyme label (Meigu Molecular Instruments, Spectramax iD2) by adding 50 μL of BacTiter GloTM reagent (Promega, G8230), which was pre-balanced at room temperature for 15 min, away from light. Three biological replicates were conducted for each sample.

### Measurement of NADH content

According to the experimental method of survival rate, 2.5 mg/mL PA was added or not. After incubation for 6 h, the bacteria were removed, and the concentration was adjusted to 440 CFU/μL. NADH was measured according to the NADH test kit (Beyotime, S0175), and the absorbance at 450 nm was measured using an enzyme label (Meigu Molecular Instruments, Spectramax M2). Three biological replicates were conducted for each sample.

### Enzyme activity assay

According to the experimental method of survival rate, 2.5 mg/mL PA was added or not, and after incubation for 6 h. The bacterial precipitate was collected and resuspended in saline adjusted to an OD_600_ of 1.0. Ten milliliters of the bacterial solution was centrifuged, the supernatant was discarded, and 1 mL of normal saline was added for re-suspension and transferred to a 1.5 mL centrifuge tube. Enzymatic activities of PDH, α-KGDH, SDH, and MDH were measured according to the instructions of the test kit (Solarbio, BC0385, BC0715, BC0955, BC1045). Three biological replicates were conducted for each sample.

### Quantitative real-time PCR analysis

Quantitative real-time PCR (qRT-PCR) was carried out and modified as described previously (46). Bacteria treated with PA for 6 h or untreated were collected according to the survival rate method and adjusted to an OD_600_ of 1.0. Each 2 mL of the above-mentioned bacteria was harvested by centrifugation at 12,000 × *g* and 4°C for 3 min and immediately quenched in liquid nitrogen. Then, the cells were lysed to extract total RNA using the bacterial RNA extraction kit (Omega, R6950-01). Reverse transcription-PCR was conducted with 1 μg of total RNA, according to the instruction manual (Promega, LS2052). Then, qRT-PCR was performed using a PrimeScript RT reaction kit (Promega, LS2062). The qRT-PCR was conducted in a 96-well plate with the following reaction components: GoTaq qRT-PCR Master Mix (2×) 5 μL, forward primer, reverse primer (10 μM) 0.2 μL, cDNA template 1 μL (diluted 20 times), and ddH_2_O to a final volume of 10 μL. Primers sequences were listed in Table S4. The reaction mixture was then subjected to amplification on a 7500 Real-Time PCR system (Applied Biosystems). The thermal cycling parameters were as follows: initial denaturation at 95°C for 2 min to activate the polymerase and pre-denature the template DNA, followed by 40 cycles of denaturation at 95°C for 15 s, annealing and extension at 60°C for 1 min. A final dissociation curve was generated by heating at 95°C for 15 s, 60°C for 15 s, and 95°C for 15 s. The data were analyzed using the software of the 7500 Real-Time PCR system, and the relative mRNA expression levels were calculated. Each sample was prepared with four biological replicates and two technical replicates.

In this experiment, a relative quantitative method was employed to analyze the data using the following formula: ΔCt = Ct (target gene) − Ct (16S rRNA gene), ΔΔCt = ΔCt (target gene) − ΔCt (control), fold change = 2^−ΔΔCt^. Quantified mRNA expression was normalized to 16S rRNA, and expression was presented relative to control levels using the 2^−ΔΔCt^ method of analysis.

### Evaluation of the effect of PA *in vivo* mouse infection model

ECO-R_AMP_ was transferred into 100 mL LB at a ratio of 1:50 and cultured at 37°C with shaking at 200 rpm until the concentration of the bacteria was OD_600_ = 1.0 ± 0.05. After centrifugation at 5,000 × *g* for 3 min, the bacteria were collected and washed twice with fresh LB, and then adjusted to an OD_600_ of 2.0 with fresh LB, and then concentrated four times to make the bacterial concentration 3.4 × 10^13^ CFU/µL. Mice were intraperitoneally injected with 60 μL of this bacterial solution. One hour later, 100 μL of 10, 20, or 30 mg/kg PA or 20 mg/kg AMP solution dissolved in fresh LB medium was intramuscularly injected (47). The survival state of mice within 7 days was observed. Humane endpoints were strictly followed to minimize animal suffering. Mice were euthanized if they met any of the following criteria: (i) weight loss of more than 20% of baseline body weight; (ii) inability to access food or water. Euthanasia was performed by cervical dislocation.

In the assessment of organ infection conditions, after 12 and 24 h of treatment, the infected mice were euthanized and dissected, kidneys, spleens, and livers were taken and weighed in a sterile centrifuge tube. Add three grinding beads and sterile saline at a ratio of 2 mL/g, and grind evenly using a high-speed and low-temperature tissue grinder (Wuhan Xavier Biotechnology Co., Ltd.). Set the temperature at 4°C, the operating frequency at 70 Hz, the running time at 30 s, the pause time at 10 s, and the number of runs at 10 (liver) or 15 (kidney, spleen) times. The homogenate was diluted with normal saline at a 10-fold ratio, and 5 μL was placed on an LB agar square plate. The bacterial content (CFU/g) in the organs was calculated as bacterial count/tissue weight.

### Cytotoxicity assay

The cytotoxicity of PA on Vero (Verda Reno) cells was evaluated using a Cell Counting Kit-8 (CCK-8) assay. Briefly, cells were seeded into 96-well plates at a density of 7 × 10³ cells per well and allowed to adhere overnight. Subsequently, the culture medium was replaced with fresh medium containing PA at 1, 0.5, 0.25, 0.125, 0.0625, and 0.03125 mg/mL. The cells were then incubated with PA for 24 h. Following the treatment, the medium was carefully removed, and each well was replenished with 100 μL of fresh medium mixed with 10 μL of CCK-8 solution. After further incubation at 37°C for 1 h, the absorbance at 450 nm was measured using a microplate reader (company: Hangzhou Aosheng Instrument Co., Ltd., China; model: Feyond-A30). Cell viability was calculated as a percentage relative to the untreated control group. All experiments were performed in at least three independent replicates.

### Statistical analyses

Statistical analysis was performed using GraphPad Prism 8.0.1. All data are presented as mean ± SD. One-way ANOVA or two-way ANOVA among multiple groups was used to calculate *P* values. Significance levels are indicated by numbers of asterisks: *P < 0.05, **P < 0.01, ***P < 0.001. All abbreviations and full names of the words are listed in Table S5.

## Data Availability

The data and materials used in this research are available from the corresponding author upon reasonable request. The whole-genome sequencing data for the ECO-R_AMP_ have been deposited in the NCBI Sequence Read Archive (SRA) under BioProject accession number PRJNA1380888. Separately, the GC-MS data have been deposited in the OMIX repository of the China National Center for Bioinformation (https://www.cncb.ac.cn/b1/) under accession number OMIX014737.

## SUPPLEMENTAL MATERIAL

Supplemental material spectrum.02879-25-s0001.docx Tables S1 to S5; Fig. S1 to S6.

ASM does not own the copyrights to Supplemental Material that may be linked to, or accessed through, an article. The authors have granted ASM a non-exclusive, world-wide license to publish the Supplemental Material files. Please contact the corresponding author directly for reuse.
