# Erratum for Cheng et al., “Metabolic activation of pyruvate cycle by protocatechualdehyde triggers oxidative killing of ampicillin-resistant *Escherichia coli”*

**DOI:** 10.1128/spectrum.01584-26

**Published:** 2026-08-25

**Authors:** Liting Cheng, Yue Liu, Yifei Ge, Shuhan Yang, Tao Yuan, Sufang Kuang

## ERRATUM

Volume 14, no. 4, e02879-25, 2026, https://doi.org/10.1128/spectrum.02879-25. Acknowledgments section and Funding table: The National Natural Science Foundation of China grant number “22207050” should read “22507050.”

