# Supplementary material for: Metabolic activation of pyruvate cycle by protocatechualdehyde triggers oxidative killing of ampicillin-resistant Escherichia coli
Source: Microbiol Spectr. 2026 Mar 18;14(4):e02879-25. doi: 10.1128/spectrum.02879-25 (PMC13055212; doi:10.1128/spectrum.02879-25)
Supplement: Supplemental material — Tables S1 to S5; Fig. S1 to S6. [file spectrum.02879-25-s0001.docx]

**Supplementary material**

**Table S1 MIC of MDR-ECO2, MDR-KPN3 and MDR-PAE2.**

| Antimicrobial agent | Cefazolin  sodium | Cefuroxime  sodium | Cefotaxime sodium | Piperacillin  sodium | Ampicillin | Tetracycline | Gentamicin | Kanamycin | Amikacin sulfate | Ofloxacin | Imipenem | Meropenem |
| --- | --- | --- | --- | --- | --- | --- | --- | --- | --- | --- | --- | --- |
| Breakpoints  (μg/mL) | R≥8 S≤2 | R≥32 S≤8 | R≥4 S≤1 | R≥32 S≤8 | R≥32 S≤8 | R≥16 S≤4 | R≥16 S≤4 | R≥16 S≤4 | R≥64 S≤16 | R≥2 S≤0.5 | R≥4 S≤1 | R≥4 S≤1 |
| MDR-ECO2 | >128 | >128 | >128 | >128 | >128 | ≥64 | >128 | ≥32 | ≤16 | ≥64 | ≥0.5 | ≥0.5 |
| MDR-KPN3 | >128 | >128 | >128 | >128 | >128 | ≥64 | >128 | ≥16 | ≤4 | ≥64 | ≥0.5 | ≥1 |
| Breakpoints  (μg/mL) | R≥8 S≤2 | R≥32 S≤8 | R≥32 S≤8 | R≥128 S≤16 | R≥32 S≤8 | R≥16 S≤4 | R≥16 S≤4 | R≥25 S≤16 | R≥64 S≤16 | R≥4 S≤1 | R≥8 S≤2 | R≥8 S≤2 |
| MDR-PAE2 | >128 | >128 | >128 | >128 | >128 | ≥64 | >128 | ≥32 | ≤8 | ≥64 | ≥0.5 | ≥1 |

**Table S2 The MIC and FIC of ECO-R_AMP_ and MDR-ECO2 were affected by the combined use of PA and antibiotics.**

| Medication | ECO-R_AMP_ MIC（μg/mL） | | FIC index | Outcome | MDR-ECO2 MIC（μg/mL） | | FIC index | Outcome |
| --- | --- | --- | --- | --- | --- | --- | --- | --- |
|  | Alone | Combination |  |  | Alone | Combination |  |  |
| Meropenem | 0.0195 | 0.0195 | 1.0156 | Irrelevant | 0.6250 | 0.3125 | 1.0000 | Irrelevant |
| PA | 800 | 12.5 |  |  | 800 | 400 |  |  |
| Balofloxacin | 2.5 | 2.5 | 1.0156 | Irrelevant | 160 | 0.156 | 0.5010 | Additive |
| PA | 800 | 12.5 |  |  | 800 | 400 |  |  |
| Cefuroxime Sodium | 10 | 10 | 1.0156 | Irrelevant | 160 | 0.3125 | 1.0020 | Irrelevant |
| PA | 800 | 12.5 |  |  | 800 | 800 |  |  |
| Amikacin | 10 | 5 | 0.5156 | Additive | 5 | 5 | 1.5000 | Irrelevant |
| PA | 800 | 12.5 |  |  | 800 | 400 |  |  |
| Micronomicin | 10 | 5 | 0.5156 | Additive | 160 | 0.625 | 0.5039 | Additive |
| PA | 800 | 12.5 |  |  | 800 | 400 |  |  |

**Table S3** **The company and the country of origin of the antibiotics.**

| Antibiotics | Company | CAS |
| --- | --- | --- |
| Cefazolin sodium | Macklin | 27164-46-1 |
| Cefuroxime sodium | Macklin | 56238-63-2 |
| Cefotaxime sodium | Macklin | 64485-93-4 |
| Piperacillin sodium | Macklin | 59703-84-3 |
| Ampicillin | Sangon Biotech | 69-52-3 |
| Tetracycline | Macklin | 60-54-8 |
| Gentamicin | Sangon Biotech | 1405-41-0 |
| Kanamycin | Sangon Biotech | 25389-94-0 |
| Amikacin sulfate | Solarbio | 39831-55-5 |
| Ofloxacin | Sangon Biotech | 82419-36-1 |
| Imipenem | Solarbio | 74431-32-5 |
| Meropenem | Solarbio | 96036-03-2 |
| Balosanide | Yuanye Biotech | 127294-70-6 |
| Minocycline | Macklin | 52093-21-7 |

Note: All companies are from China.

**Table S4** qRT-PCR Primer Sequence

| Primer | F | R |
| --- | --- | --- |
| *16S rRNA* | GTAGTCCACGCTGTAAACGA | GAATTAAACCACATGCTCCA |
| *aceE* | AGGTCGTCTGACTCAGGAGC | CCATAGATACGGTCGGGAAC |
| *aceF* | CCGAGTTGGAAGCGTT | TGCCTTTCTTGTTGACG |
| *lpd* | CGTCGGCTGTATCCCTT | AGACCGTTGACCACTTTGA |
| *gltA* | CAAAAGCAAAACTCACCCT | GCAGCAAAATACCTTCATCAC |
| *prpC* | CGGGGCGAATGAAGTGT | GGGTCAGCGATGGTGTAAA |
| *acnA* | GAAGCCGCAATGTTAG | ACGGTAGTGAATCCAGAC |
| *acnB* | TGGTTTGTGCCTCGG | TCGCCTGACGGAACACA |
| *icd* | GAGATGGGGGTGAAGAAAA | CTGCCAGGGCGTCAGAAAT |
| *sucA* | GCTGGAGCAGGAAAAAGT | TGGCGGTGGAAGAAGGTA |
| *sucB* | GAAGAGCAAAACAACGATG | AGTCAGACGACCACCCA |
| *sucC* | CGGCGGTATCGTTCGTT | GCATCCGTCAGACCTTTT |
| *sucD* | AAGCCGTTGCTGCCACT | GACCGAAACCGTAATCC |
| *sdhA* | AATGAGAAAGGCGAAGATG | CACGGAAGACCGAGAAG |
| *sdhB* | ACGGGCTGTATGAATGT | TCGGTATCACGGCTATC |
| *sdhC* | CTGCTGTGGCTTCTGGGTA | TTGGCGGAGCGTTTA |
| *sdhD* | TAAGCAACGCCTCCG | ACACCCCACACCACAAC |
| *frdA* | CTCAAACACACCCTCGC | GCTGCTTCCGCCTTAT |
| *frdB* | ACTAACATCCAGACCCCG | CGCTCCTTCTTACCGTG |
| *frdC* | AACTGGCACCGAAAGC | GGGCAACAAACAGGATT |
| *frdD* | CGTATTCCTGTTCCTGAT | GACCGTAGAAAACCCATT |
| *fumA* | TGGCGACGAATACAAAT | TCCACCAATAACGAACG |
| *fumB* | AACCGCCCGATGAAA | AAGCGACGCACTCCA |
| *fumC* | ATGGGGGCGATTGATG | GTTGGAACTTTGGCTTTTGT |
| *fumD* | GGACAAAAGAGGATGAAC | AATACGCCTGCGATAA |
| *fumE* | AACGAACAGGATGAAACG | ACGGAGAAATCAGCGAA |
| *mdh* | GTTACCATTCTGCCGCT | GATAGATTTACGCTCTTCCAC |
| *mqo* | CAATGGCAGGCTACG | CTTCTTTTTGAACGGGT |
| *maeB* | AATCGCCTGTATGAACCTGCT | CCCTGATAGATAACGCCTTTTG |
| *pflB* | AACGCACTGGGTAAAGACGA | GCATTTCACGGTTCATCACG |
| *tdcE* | GCAACGCCGTCCCTACC | CAGCATTTCCCGATTCATTA |

**Table S5** Abbreviation table

| Abbreviation | Full name |
| --- | --- |
| AMDIS | Automated mass spectral deconvolution and identification system |
| AMP | Ampicillin |
| CARSS | China antimicrobial resistance surveillance system |
| CFU | Colony-forming units |
| CLSI | Clinical & laboratory standards institute |
| *E. coli* | *Escherichia coli* |
| ECO2 | Escherichia coli 2 |
| ECO-R_AMP_ | Ampicillin resistant Escherichia coli |
| ECO-S | Susceptible Escherichia coli |
| EI | Electron ionization |
| EI-MS | Electron ionization mass spectrometry |
| GC-MS | Gas Chromatography-Mass Spectrometry |
| HPLC | High performance liquid chromatography‌ |
| KPN3 | *Klebsiella pneumoniae* 3 |
| LB | Luria-Bertani |
| MDH | Alate dehydrogenase |
| MDR | Multi-drug resistant |
| MHB | Mueller-hinton base |
| MIC | Minimal inhibit concentration |
| MRSA | *Staphylococcus aureus* |
| NAC | N-acetylcysteine |
| NIST | National institute of standards and technology |
| PA | Protocatechualdehyde |
| PAE2 | *Pseudomonas aeruginosa* 2 |
| PDH | Pyruvate dehydrogenase |
| qRT-PCR | Quantitative real-time PCR |
| ROS | Reactive oxygen species |
| SDH | Succinate dehydrogenase |
| SEM | Scanning electron microscope |
| TCA | Tricarboxylic acid cycle |
| α-KGDH | α-Ketoglutarate dehydrogenase |


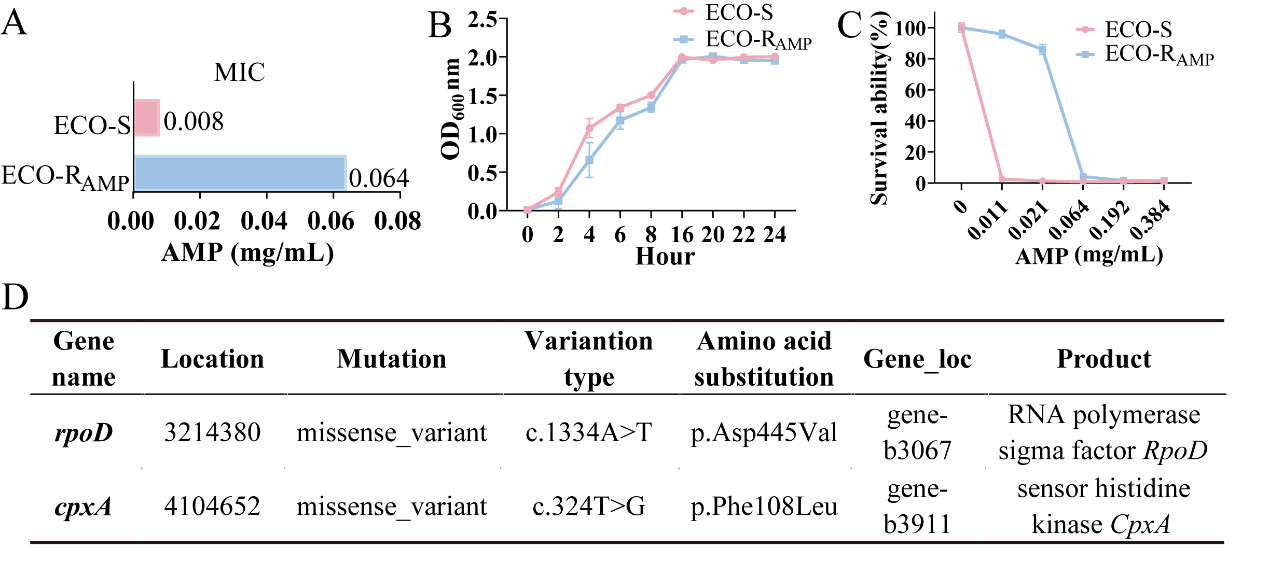


**FIG S1. Phenotypic analysis of** **artificial passage ampicillin-resistant bacteria (ECO-R_AMP_). (A)** MIC determination of AMP to *sensitive Escherichia coli* (ECO-S) and ECO-R_AMP_. (B) **Growth curve assessment of ECO-S and ECO-R_AMP_. (C)** **Survival ability measurement of ECO-S and ECO-R_AMP_ with AMP. (D)** **The mutation site of ECO-R_AMP_.**

**
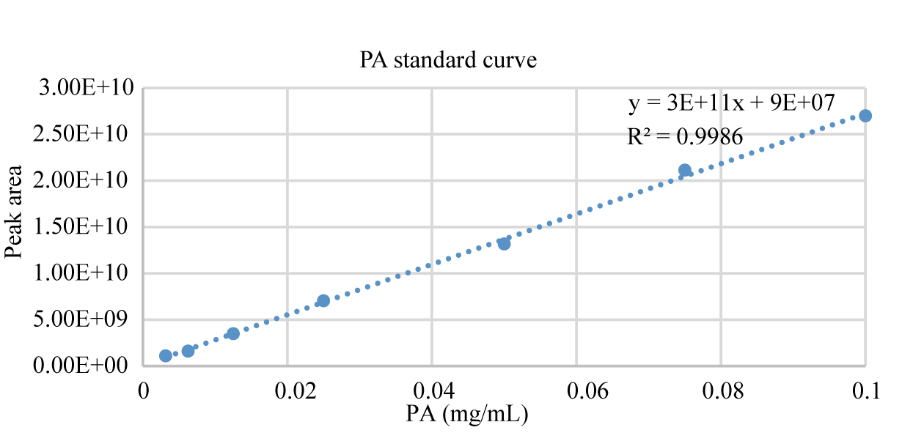
**

**FIG S2.** Standard curve of PA.

**
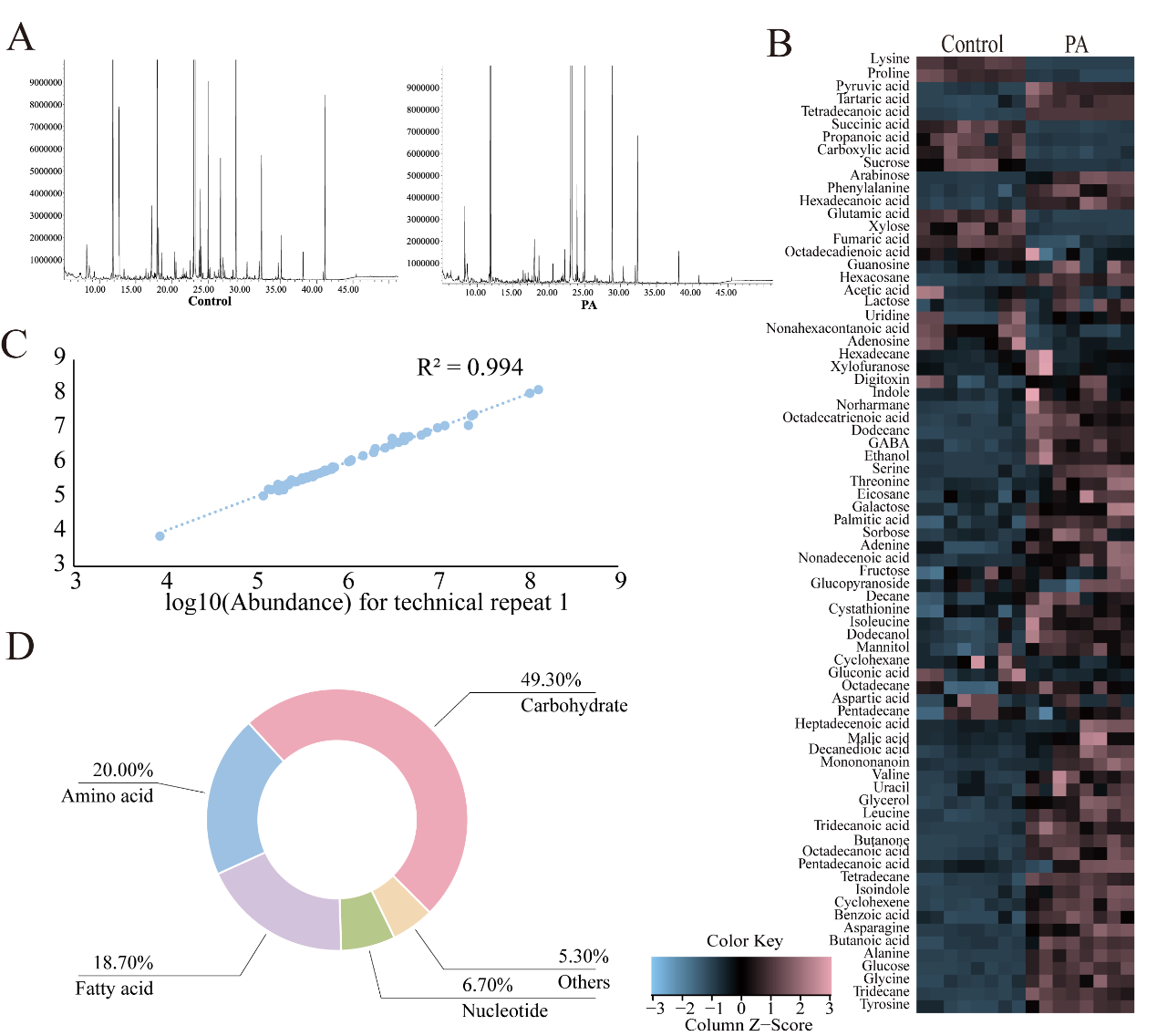
**

**FIG S3. Metabolic profiles analysis in ECO-R_AMP_** **with or without PA.** (A). Representative total ion chromatograms of metabolic abundance in ECO-R_AMP_ and PA-treated resistant bacteria (ECO-R_AMP_-PA). (B) Heat map of unsupervised hierarchical clustering of all metabolites (row). Red and blue colours indicate increase and decrease of the metabolites scaled to mean and standard deviation of row metabolite level, respectively (see colour scale). (C) Pearson correlation coefficient between technical replicates. (D) Categories of all of the identified metabolites.

**
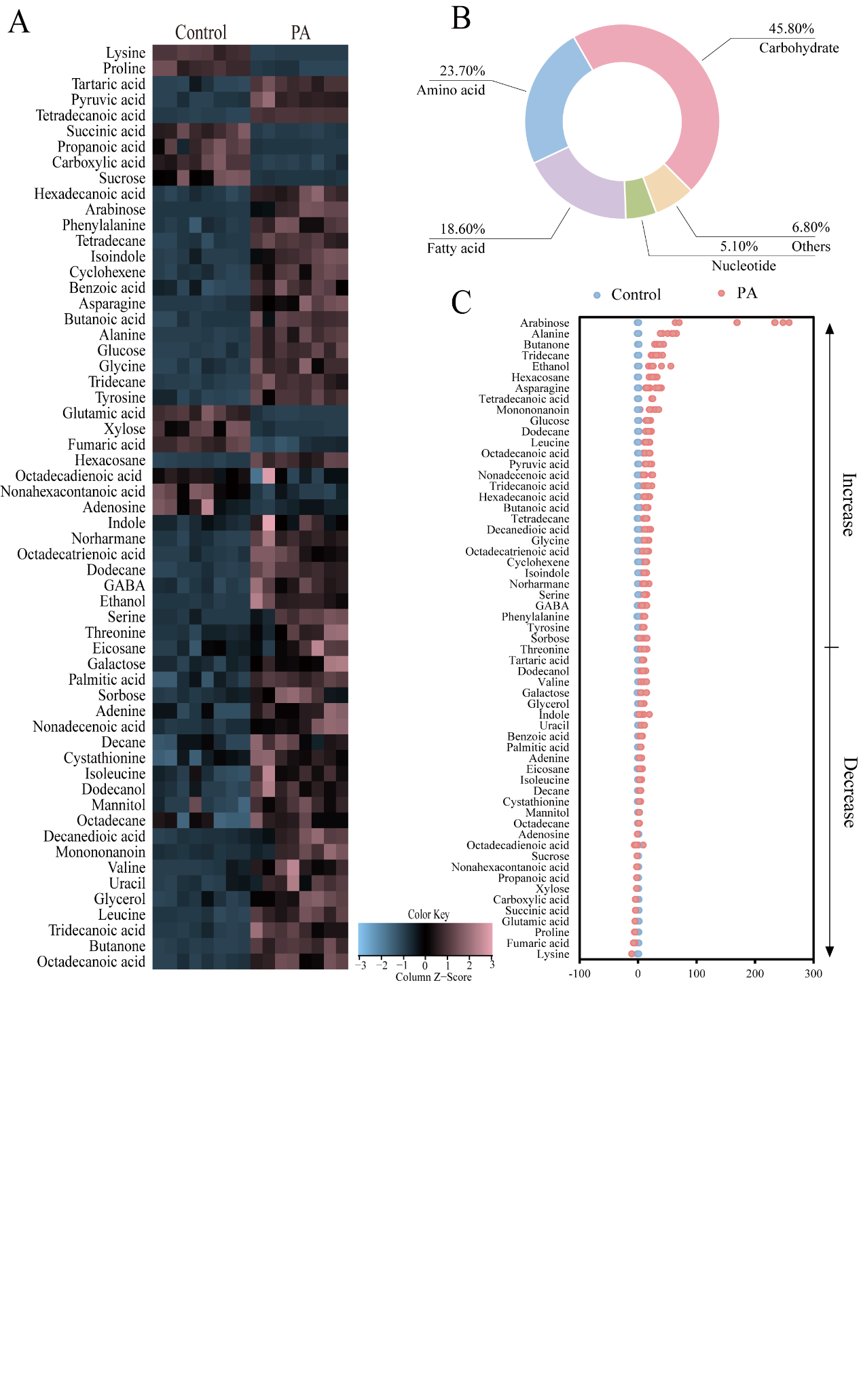
FIG S4. Differential metabolomic phenotype analysis in ECO-R_AMP_ with or without PA.** (A) Unsupervised hierarchical clustering of differential metabolic heatmap of ECO-R_AMP_ without PA (control) versus ECO-R_AMP_ with PA by row. Color scale from blue to red correlates with maximum depletion to maximum enrichment of each metabolite. (B) The categories of all identified differential metabolites. (C) Z-score plot of differential metabolites based on control.


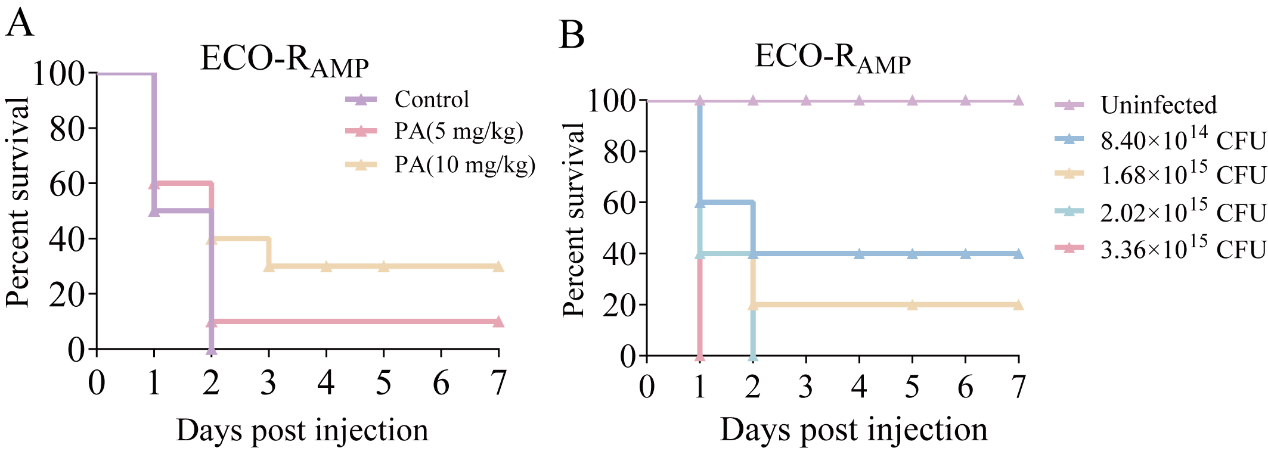


**FIG S5.** **Survival ability of mice** infected with ECO-R_AMP_. (A) The effect of low-dose PA on the survival ability of infected mice. (B) Survival rate of infected mice without any treatment. The body weight of all the tested mice was 20±2 g, groups in (A) and (B) used 10 and 5 mice, respectively.


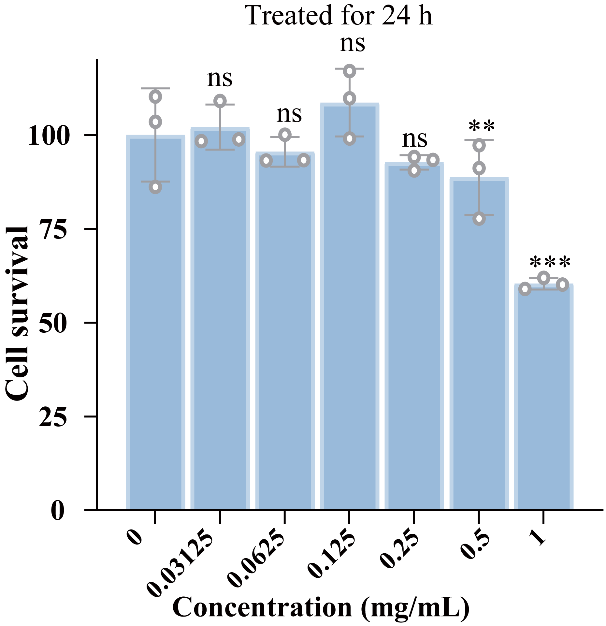


**FIG S6.** **Cytotoxicity determination of PA.**
